# Shortcutting Photorespiration Protects Potato Photosynthesis and Tuber Yield Against Heatwave Stress

**DOI:** 10.1111/gcb.17595

**Published:** 2024-12-04

**Authors:** Katherine Meacham‐Hensold, Amanda P. Cavanagh, Peyton Sorensen, Paul F. South, Jessica Fowler, Ryan Boyd, Jooyeon Jeong, Steven Burgess, Samantha Stutz, Ryan N. Dilger, Moonsub Lee, Nicholas Ferrari, Justin Larkin, Donald R. Ort

**Affiliations:** ^1^ Carl R Woese Institute for Genomic Biology University of Illinois Urbana Champaign Urbana Illinois USA; ^2^ School of Life Sciences University of Essex Colchester UK; ^3^ Department of Crop Sciences University of Illinois Urbana Illinois USA; ^4^ Department of Biological Sciences Louisiana State University Baton Rouge Louisiana USA; ^5^ Department of Plant Biology University of Illinois Urbana Illinois USA; ^6^ Department of Animal Sciences, Division of Nutritional Sciences, Neuroscience Program University of Illinois Urbana Illinois USA; ^7^ Chungbuk National University Cheongju Republic of Korea

**Keywords:** climate change, food security, photorespiration, photosynthesis, thermotolerance

## Abstract

Over two growing seasons, a chloroplast localized synthetic glycolate metabolic pathway expressed in potato, enhanced tuber biomass. We confirmed that this yield benefit did not come at the cost of tuber quality. In 2022, after two early season natural heatwaves, we observed enhanced daily carbon assimilation rates and increased photosynthetic capacity, with transformed plants having up to 23% higher *V*_cmax_ and 13% higher *J*_max_ during tuber bulking stages, indicating that transformed plants were better able to withstand growing season heatwaves than untransformed controls. The increases in photosynthetic capacity and potato tuber mass after early season heatwaves were greater than in seasons without heatwaves and present the AP3 pathway as a promising avenue for yield increases in the face of forecast increased intensity and duration of heatwave events as a result of global warming.

## Graphical abstract

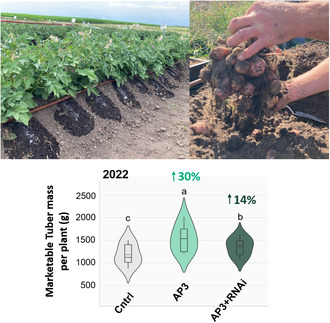

## 1 Introduction

Global food insecurity has continued to rise (WHO 2020) in tandem with plateauing yield increases for key global food crops (Ray et al. 2013). The recent coronavirus pandemic further exacerbated global food supply constraints (Panghal et al. 2022) spotlighting the pressing need for improved domestic and subsistence agricultural production of key food crops to ensure local food security for those at greatest risk. Compounding the challenge, climate predictions forecast rising global mean temperatures and increased frequency, duration, and intensity of heatwave events that could be further detrimental to crop yields (Breshears et al. 2021; Hatfield and Prueger 2015; Perkins, Alexander, and Nairn 2012). To sustainably meet food demand, crop yields must increase per unit land area without proportional increase in inputs and greenhouse gas emissions. Improving photosynthesis together with thermal tolerance offers avenues to meet this challenge (Long, Marshall‐Colon, and Zhu 2015; Ort et al. 2015; Simkin, López‐Calcagno, and Raines 2019).

Photorespiration has been identified as a process that reduces photosynthetic carbon fixation in C3 cereal crops by 20%–50% (Walker et al. 2016) making it a promising target for improving photosynthetic efficiency, heat stress tolerance, and crop yield (Betti et al. 2016; Hagemann and Bauwe 2016; Peterhansel et al. 2013; South et al. 2018). Photosynthetic carbon fixation in plants occurs with the reaction of Ribulose‐1,5‐bisphosphate (RuBP) and CO_2_ catalyzed by the enzyme Rubisco. However, Rubisco can also fix O_2_ rather than CO_2_ to RuBP, forming phosphoglycolate, which is converted to glycolate, initiating the photorespiratory pathway. Photorespiration is energy‐expensive and results in a net loss of fixed carbon (Bauwe, Hagemann, and Fernie 2010; Ogren 1984). In dry and hot conditions, RuBP oxygenation occurs more frequently due to Rubisco's higher affinity for O_2_ as temperatures increase (Badger and Andrews 1974; Jordan and Ogren 1984). Thus, photorespiratory‐linked losses in productivity are heat sensitive and are likely to have greater impact in geographic regions facing climate extremes, which are already among the most food insecure (Cavanagh and Ort 2023).

The introduction of various photorespiratory bypasses has been shown to increase plant biomass in a range of species (Kebeish et al. 2007; Maier et al. 2012; Peterhansel et al. 2013; Peterhansel and Maurino 2011). Although most of these have been demonstrated in model plants, the expression of glycolate dehydrogenase in potato by Nölke et al. (2014) and the introduction of cyanobacterial photorespiratory glycolate catabolism into potato plants by Ahmad et al. (2016), both of which increased plant biomass and photosynthetic performance, demonstrate the potential of this strategy for increasing yield of agronomically important crops. However, their role in conferring thermotolerance has only been explored in model plants (Cavanagh et al. 2022).

**FIGURE 1:**
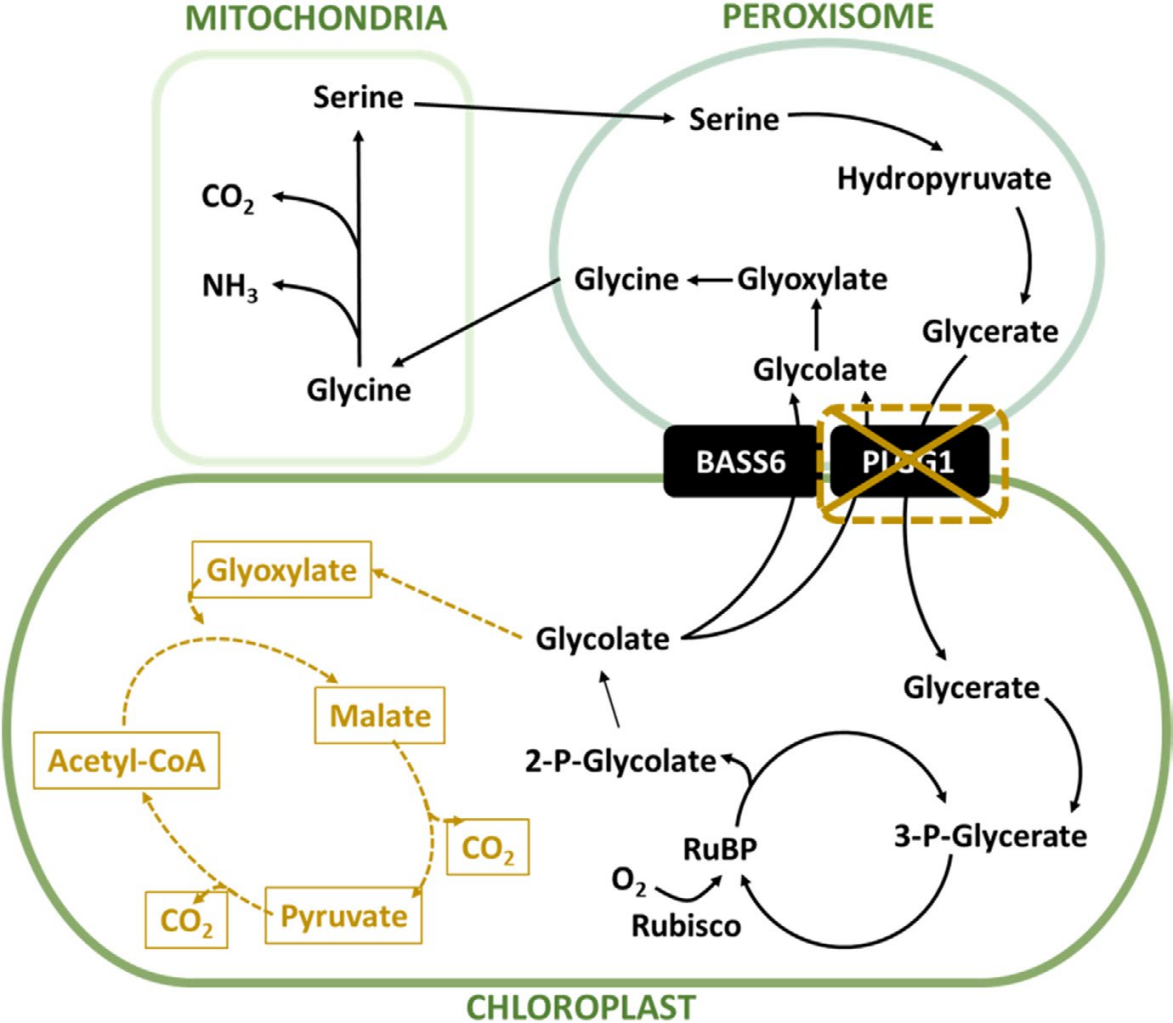
The native and introduced alternative photorespiratory pathway (AP3).  The native photorespiratory metabolism pathway through the chloroplast, peroxisome, and mitochondria is represented in solid black lines. The introduced AP3 chloroplastic glycolate metabolism pathway is shown in gold dashed lines. In the AP3 + RNAi pathway, the knocked down native glycolate–glycerate transporter PLGG1 reduces flux through the native pathway represented by the gold x.

Recently, we engineered an alternative photorespiratory pathway (AP3) that demonstrated enhanced photosynthetic performance and large increases in plant biomass in field experiments in model species *Nicotiana tabacum* (South et al. 2019) and our subsequent field study with elevated temperature treatments confirmed an enhanced thermotolerance in tobacco plants expressing AP3 (Cavanagh et al. 2022). As such we implemented AP3, described in South et al. (2019) (Figure 1) into potato ( *Solanum tuberosum* ). In terms of global production, potato is the most important non‐grain crop and the fourth most highly produced crop behind maize, wheat, and rice (FAO 2021) and shares with maize and rice the highest production of calories/land area (Poore and Nemecek 2018). It is also likely that species with vegetative storage will show greater potential for yield increase compared with grain crops, given the enhanced carbon sink capacity, increasing the possibility for increased CO_2_ fixation to translate to yield gains. Grain crops are known to suffer a detriment from heatwave events at reproduction (Ferguson et al. 2021; Siebers et al. 2017; Templ and Calanca 2020) that may be offset in tuber and storage root crops.

In this study, we hypothesized that field grown potato tuber mass would increase as a result of the introduction of AP3, due to reduced photorespiratory carbon losses and increased heatwave thermostability. Over two growing seasons, we tested and carried out single location field trials, which indicated that AP3 increased tuber biomass, in one year driven to the highest yield advantage by thermoprotection to early season heatwave events. The nutritional quality of potato tubers was not impacted.

## 2 Methods

### 2.1 Construct Design and Genetic Transformation of Plants

*Solanum tuberosum* cv. Desiree was genetically transformed to contain the AP3 photorespiratory pathway outlined in Paul F. South et al. (2019) with *Chlamydomonas reinhardtii* glycolate dehydrogenase (CrGDH), *Cucurbita maxima* malate synthase (CmMS), and RNAi targeting the glycolate–glycerate transporter *PLGG1* (Figure 1). Control plants had been through the transformation protocol, but did not contain the transgenic construct. Transformation was performed by BTI *Agrobacterium tumefaciens* strain 3C5ZR with kanamycin selection according to Van Eck et al. (2007). Plasmids schematics are depicted in Figure S1.

The transformation generated six AP3 and nine AP3 + RNAi lines, verified by qPCR to show the presence of the introduced genes and qRT‐PCR to determine the levels of gene expression. Three independent transformation events of each construct were taken forward for preliminary field testing in 2019.

### 2.2 Plant Growth

#### 2.2.1 2019

Three AP3 lines (36030‐6, 36030‐9, 36030‐14), three AP3 + RNAi lines (36031‐5, 36031‐7, 36031‐17), and two independent untransformed controls were selected for preliminary field trial testing. For field transplant, plants were propagated from tubers. On May 8, 2019, tubers were quartered, chitted, transplanted to 1 gal. pots in potting soil, and kept in growth chamber conditions on a 22°C/16 h light 15°C/8 h dark cycle, 60% RH for 3 weeks. After 3 weeks, plants were moved to greenhouse conditions (natural light 22°C day/15°C night) for 7 days, then hand transplanted to the field site on June 4, 2019 in a randomized block design using three replicate blocks (Figure S3a). Each plot contained ten plants per line per row with 10″ spacing between each plant, 36″ between rows, and 20″ between lines in a row.

#### 2.2.2 2020

In 2020, the highest yielding AP3 (36030‐9) and AP3 + RNAi (36031‐17) transformation events from 2019 and two controls (Cntrl1 and Cntrl2) were selected for field analysis. Due to uneven plant establishment in 2019, in 2020, plants were propagated from tissue culture plantlets grown in vitro rather than from tubers to ensure homogenous establishment. For propagation, plantlets were divided into nodal cuttings sectioned from a single shoot according to Roca et al. (1978) and placed in MS growth media (Murashige and Skoog 1962). Cuttings were kept in a walk‐in growth chamber at 20°C/16 h day, 18°C/8 h night at 50% relative humidity, and daytime light intensity of 700 μmol m^−2^ s^−1^.

At 8 weeks after tissue culture division, plantlets were transplanted into peat pots in potting soil, and kept in growth chamber (Conviron) conditions 22°C/14 h day, 18°C/10 h night at 700 μmol m^−2^ s^−1^ daytime light intensity, and 50% RH for 12 days. Five days prior to field planting, plantlets were moved into the greenhouse for acclimation to diurnal solar conditions (26°C/14 h day, 20°C/10 h night) (Figure S9) before being transplanted to the field.

Plants were transplanted to the field on July 1st in a complete randomized block design with six replicate plots of each event (Figure S3b). Plants were sown 12 in. apart in a single row of 10 plants per plot with 20 in. spacing between lines in a single row and 36 in. between rows. Throughout growth, plants were hilled (soil mounded to cover lower leaves about 20 cm from ground level) to promote tuber growth four times at 10, 25, 40, and 55 days after transplant to field (DAT) (Pictured in Figure S10). Plots were watered as needed between 8:00 and 09:00 h through drip irrigation installed on either side of each hill (pictured in Figure S11).

#### 2.2.3 2021

In 2021, the same plant tissue culture propagation and establishment protocol were followed as in 2020, but the consistently poorer performing control (Cntrl 2) was dropped from the experiment to allow increased repetition, leaving only Cntrl 1. Plantlets were transplanted to the field on June 18, 2021 in a complete randomized block design with eight replicate plots of each event, AP3 (36030‐9), AP3 + RNAi (36031‐17), and Cntrl 1 (Figure S3c). Each plot was spaced with the same dimensions as in 2020. At 8 days after field transplant, the experiment was destroyed by a flooding event.

#### 2.2.4 2022

The 2021 protocol and field plan (Figure S3c) was followed to repeat the flooded experiment, with plants transplanted to the field on May 23, 2022. Mounding took place at 10, 25, and 40 days after transplant (as in Figure S10). Plots were watered as needed between 8:00 and 09:00 h through drip irrigation on both sides of each hill in each plot (pictured in Figure S11).

All field trials were carried out at the University of Illinois Energy Farm Facility in Urbana, Illinois (40°03′46.4″ N 88°12′25.4″ W, 215 m above sea level).

### 2.3 Gene Expression Analysis

In 2019, 100 mg of leaf material was harvested from three plants per plot, flash frozen in liquid nitrogen, and stored at −80 °C. RNA was extracted using the Nucleospin RNA kit (Macherey‐Nagal GmbH& Co.KG, Düren, Germany). cDNA was generated using the Qiagen Quantinova reverse transcription kit. Gene expression analysis was performed on a Bio‐Rad CFX real‐time PCR system. Relative changes in gene expression were determined using reference genes *L25 and Eif1a* with three technical replicates per biological sample. Amplification was performed using the Bio‐Rad SSO advanced SYBR green master mix, and relative levels were determined using the ΔΔCT method.

In 2020 and 2022, at 21 days after transplant, leaf tissue was collected from the last fully expanded leaf of each plant for total RNA extraction. A single hole punch from each plant in a plot, totaling ten leaf disks, which was approximately 100 mg. Tissue was collected from every plot in the field experiment. The tissue was immediately flash frozen in liquid nitrogen and stored at −80°C. Total RNA was extracted with the RNeasy Plant Mini Kit (74904: Qiagen) and treated with DNase I (79254: Qiagen) to remove gDNA. Qubit RNA IQ Assay Kit was used (Q33221) to ensure RNA had sufficient integrity and quality, with an IQ score greater than 9/10 before cDNA synthesis. The RNA was reverse‐transcribed using the SuperScript III First‐Strand Synthesis System (18080051; Invitrogen). An additional nonreverse transcriptase (NRT) control was made for each RNA sample by following the same protocol but replacing the SSIII enzyme with water. The cDNA was then diluted to 5 ng/μL. A qPCR run was conducted to ensure there was minimal genomic DNA contamination by comparing Cq values of synthesized cDNA and the NRT control. Each sample was determined to have a difference in Cq values of at least 5 between its corresponding NRT control and cDNA.

Real‐time quantitative PCR (RT‐qPCR) assays were conducted on the CFX Connect real‐time system (Bio‐Rad) using SsoAdvanced Universal SYBR Green Supermix (1725270: Bio‐Rad) to detect two reference genes, *cyclophilin (cyc)* and *tubulin* (*tub*) and three genes of interest (CrGDH, CmMS, and PLGG1). The software qbase+ (Biogazelle) was used to calculate relative gene expression from the Cq values by employing a generalized delta–delta‐Ct model according to Hellemans et al. (2007). Normalized relative quantities (NRQ) were calculated for each sample using the average Cq value for its technical replicates, normalized factors that were calculated based on the reference genes, as well as amplification efficiencies calculated for each primer set.

Primers used in this work are listed in Table S1.

### 2.4 Photosynthetic Measurements

#### 2.4.1 Diurnal CO_2_ Assimilation

Diurnal CO_2_ assimilation was measured in 2022 only. On June 14, 2022 and June 21, 2022, diurnal measurements of photosynthesis began at 8:00 h until around 18:30 h, with measurement sets throughout the day commencing at 8:00, 10:00, 11:30, 13:00, 14:00, 15:30, 16:45, and 18:00 h. On June 14th, only data after 13:00 h are presented due to intermittent morning cloud cover. Snapshot measurements were made on the last fully expanded terminal leaflet on the main stem on three plants per plot (*n* = 8 with 3 subsamples per plot), with Li‐6800 (LI‐COR, Lincoln NE). Light levels inside the measuring cuvette were set to match ambient using the PPFD sensor on the Li6800. Reference CO_2_ was set to match ambient at 420 ppm for all measurements. Between June 14th and June 21st, a new leaflet had emerged and measurements were made on a new last fully expanded leaf on June 21st.

#### 2.4.2 A/Ci Response Curves

The response of assimilation (*A)* to intercellular CO_2_ partial pressure (*C*_i_) was measured on the last fully expanded terminal leaflet on the main stem with Li‐6800 in both 2020 and 2022 field experiments. In both years, curves were made at a saturating light intensity of 1800 μmol mol^−2^ s^−1^. Measurements of A began at ambient 420 μmol mol^−1^ CO_2_ to create a response curve at the following CO_2_ concentrations: 400, 300, 200, 150, 100, 75, 50, 25, 400, 400, 700, 1000, 1200, 1500, 1800, 2000 μmol mol^−1^_._ *V*_cmax_ and *J*_max_ were calculated using the C_3_ photosynthesis biochemical model of Farquhar, Caemmerer, and Berry (1980) using the curve fitting utility from Sharkey et al. (2007). In 2020, curves were made in a single campaign over 2 days, August 5th–6th (35–36 DAT, early tuber bulking) at 25°C between 8:00 and 14:00 h. In 2022, curves were made in two separate campaigns, the first on June 22nd during vegetative growth measured at 28°C and the second on July 18th during mid tuber bulking at 30°C, both between 8:00 and 14:00 h. In 2020, curves were measured in three plants per plot where *n* = 6 plots (18 subsamples). In 2022, two plants per plot were measured where *n* = 8 plots with two samples per plot (16 subsamples).

Γ (CO_2_ compensation point) was calculated from the initial slope of A/Ci response curves at Ci values below 200 μmol mol^−2^ s^−1^ as the x intercept (von Caemmerer 2000).

#### 2.4.3 A/Q Response Curves

The response of photosynthesis to light (A/Q) was measured in 2020 and 2022. In 2020, A/Q curves were made during the gas exchange campaign Aug 5th–6th, after the completion of an A/Ci curve on a given plant, leaves were stabilized at 400 μmol mol^−1^ CO_2_ and saturating light intensity 1800 μmol m^−2^ s^−1^_._ The response of A–Q was then measured at the following light levels: 1800, 1500, 1000, 400, 200, 150, 100, 75, 50, 30, 20, 10, 0 μmol m^−2^ s^−1^_._ Temperature exchange point was set to 25°C for curve duration.

In 2022, measurements were made in a single campaign over 2 days, June 28th and 29th (36–37 DAT). A/Q curves were made independently and did not follow A/Ci curves on a given leaf. The last fully expanded terminal leaflet on the main stem was stabilized at 420 μmol mol^−1^ CO_2_ and saturating light intensity 1800 μmol m^−2^ s^−1^ inside the measuring cuvette. A/Q was then measured at the following light levels: 1800, 1500, 1000, 700, 400, 200, 150, 100, 75, 50, 30, 20, 0 μmol m^−2^ s^−1^ with temperature exchange point was set to 28°C for curve duration. For all A/Q curves in both years, the minimum wait time between logging at a new light level was 180 s. For all curves, a saturating pulse was made at each data log to capture chlorophyll fluorescence dynamics.

From A/Q curves, the quantum yield of CO_2_ fixation (ɸCO_2_) was calculated from the slope of the relationship between A/Q below 100 μmol m^−2^ s^−1^ absorbed irradiance. Quantum yield of PSII (ɸPSII) was calculated from the slope of the relationship between ɸPSII/Q below 100 μmol m^−2^ s^−1^ absorbed irradiance. In both years, leaf absorption was calculated on the last fully expanded terminal leaflet on three plants per plot, as the average of three measurements per leaf, where measured absorption = 1‐(transmittance+reflectance) with a Jaz spectrometer (Ocean Optics).

#### 2.4.4 Intrinsic Water Use Efficiency (iWUE)

iWUE was calculated at the leaf level from diurnal photosynthetic gas exchange measurements as the ratio of net photosynthetic assimilation (A_n_) to stomatal conductance (g_s_) according to Leakey et al. (2019).

#### 2.4.5 Isotopic Gas Exchange and Mesophyll Conductance

Fully expanded potato leaves, exposed to full light were cut between 05:00 and 05:30 h from 3 to 13 August 2020 (34–43 DAT). Cut leaves were immediately placed in a bucket of water before being re‐cut underwater and taken to the lab where they remained in the dark until photosynthesis was measured. Leaves were placed in a growth cabinet (Gen 1000, Conviron) with an irradiance of ~700 μmol m^−2^ s^−1^, set to 25°C for approximately 5 min before the terminal leaflet was placed in the multiphase flash fluorometer chamber of the LI‐6800 (LI‐6800 Biosciences, Lincoln, NE, USA) located in the growth cabinet. Leaf temperature was set to 25°C, irradiance 1800 μmol m^−2^ s^−1^, CO_2_ sample 400 μmol mol^−1^, chamber relative humidity 60%, and an [O_2_] of either 20.9 kPa or 1.99 kPa. Leaves acclimated in the chamber for between 20 and 35 min before photosynthesis stabilized. Photosynthesis and mesophyll conductance were estimated under steady‐state conditions for approximately 30 min before the [O_2_] was changed and the leaf was allowed to acclimate for 10–15 min to the other [O_2_] before photosynthesis and mesophyll conductance were measured for approximately 30 min. The order of the [O_2_] was random. The LI‐6800 was coupled to a tunable diode laser absorption spectroscope (TDL—model TGA 200A, Campbell Scientific) that measures ^12^CO_2_ and ^13^CO_2_ (Bowling et al. 2003). The TDL setup and calibration are described in Tazoe et al. (2011). The TDL measured each site for 20 s. The LI‐6800 was set to auto log every 180 s, once per TDL measurement cycle. The TDL was connected to the LI‐6800 reference and sample using the reference and sample ports on the back of the LI‐6800 head, respectively. Photosynthetic discrimination (Δ^13^C_obs_) and mesophyll conductance (g_m_) including the ternary effect (Farquhar and Cernusak 2012) were calculated according to Evans et al. (1986) and Evans and von Caemmerer (2013).

### 2.5 Tuber Harvest

All tuber harvests were conducted by hand in a single day. Above‐ground biomass was removed, tubers were dug up and separated from roots, cleaned, counted, and weighed on a per plot basis (Figure S12). In 2019, harvest took place on October 9, 2019. In 2020, harvest took place on October 15th. In 2019 and 2020, tubers < 2 g were not included in tuber biomass totals. In 2022, harvest took place on September 14th and tubers < 5 g were counted, weighed and subtracted from the total per plot to give total marketable tuber mass per plot.

### 2.6 Tuber Nutritional Content

In 2020, three subsamples from each plot were analyzed for nutrient content, *n* = 6 with three subsamples per repetition per genotype. Immediately after tuber harvest, 500 g of tubers between 55 and 65 g each were selected for each subsample (1.5 kg per plot). Unpeeled tubers were washed, dried, and ground. Unpeeled tuber dry matter (DM) and organic matter (OM)/ash were determined according to AOAC official methods 934.01, (2006) and 942.05 (2006), respectively. Total starch was determined according to AOAC Official Method 979.10 (2006) and total dietary fiber (TDF) following AOAC Official Method 985.29 (2006). Free sugars were quantified according to Campbell et al. (1997). Total crude protein (CP), minerals, and amino acids were analyzed at Agricultural Experiment Station Chemical Lab (University of Missouri‐Columbia) all according to 2006 AOAC Official Methods as follows: AOAC method 990.03 for Crude Protein by combustion, 985.01 (a,b,d) for minerals calcium and potassium and iron using inductively coupled plasma (ICP) atomic emission spectroscopy, 982.30 E (a,b,c) for amino acids and 988.15 for Tryptophan by alkaline hydrolysis. Vitamin C and B6 were analyzed at Eurofins according to AOAC Official Method 967.22, 2006 for vitamin C and J. AOAC, 88:30–37, 2005 for Vitamin B6.

From 2020 analysis, significant differences were observed between transformed plants and controls for TDF, iron, and vitamin C. As such, these three assays were repeated from 2022 grown tubers, from two subsamples per plot (*n* = 8, 16 subsamples per line), following the same protocol used in 2020.

### 2.7 Soil Composition Analysis

Soil analysis was completed fee for service by KSI Laboratories (Shelbyville, IL, USA) on samples taken on June 23, 2022, at 32 DAT. Four soil cores were extracted from various positions in every plot and combined prior to analysis. pH tests were performed with electrode readings in 10 g of soil and 10 mL of distilled water and buffer pH tested using the Sikora buffer method (Sikora 2006). Phosphorus, potassium, calcium, magnesium, sulfur, boron, zinc, copper, iron, manganese, sodium were determined using ICP spectroscopy, using Mehlich 3 extraction (Mehlich 1978). Organic Matter was determined colormetrically (Walkley and Black 1934).

### 2.8 Leaf Photorespiratory Metabolite Analysis

To assess the response of leaf photorespiratory metabolite pool sizes, plants from AP3 line 36030–9, AP3 + RNAi line 36031–17, and control 1 were grown in growth chamber conditions where *n* = 8 plants per line. Chamber conditions were set to simulate field temperature and humidity conditions observed during 2022 field growing season, including two early season heat waves (Chamber settings in Table S5). In heat wave 1, plants were exposed to daily highs of 37°C over 4 days, and in heat wave 2, to daily highs of 37°C over 3 days.

#### 2.8.1 Mass Spectrometry

~100 mg of fresh leaf tissue was harvested from the last fully expanded terminal leaflet from each plant in mid‐afternoon (between 15:00—16:00 h) on day 4 of simulated heat wave 1. Tissue was immediately flash frozen in liquid nitrogen. Metabolite analysis was performed by the Carver Metabolomics Core, University of Illinois at Urbana‐Champaign Roy J. Carver Biotechnology Center. Metabolites were analyzed using a GC–MS system (Agilent) consisting of an Agilent 7890 gas chromatograph, an Agilent 5975 mass selective detector, and a HP 7683B autosampler. Gas chromatography was performed on a ZB‐5MS capillary column (60‐m × 0.32‐mm i.d. and 0.25‐μm film thickness; Phenomenex). The inlet and MS interface temperatures were 250°C, and the ion source temperature was adjusted to 230°C. An aliquot of 1 μL was injected with the split ratio of 10:1. The helium carrier gas was kept at a constant flow rate of 2 mL/min. The temperature program was a 5‐min isothermal heating at 70°C, followed by an oven temperature increase of 5°C min − 1 to 310°C and a final 10 min at 310°C. The mass spectrometer was operated in positive electron impact mode at 69.9 eV ionization energy at m/z 30 to 800 scan range. To allow comparison among samples, all data were normalized to the internal standard in each chromatogram and the sample wet weight. The spectra of all chromatogram peaks were evaluated using the AMDIS 2.71 program (NIST).

### 2.9 Statistical Analysis

Field experiments were analyzed from a complete randomized block design with a linear model ANOVA where *y* = *μ* + Block + Line + *ε* with a = 0.05. All data were separated by year. Line was considered as a fixed effect and block was analyzed as a random effect.

For qPCR *n* = 6 in 2020, *n* = 8 in 2022 with three technical reps per qPCR run using two tailed *t*‐tests against the Control (AP3), to compare gene expression of each transgenic against the control independently. For photosynthetic A/Ci and A/Q curves, in 2020, *n* = 6 with 3 subsamples per plot. In 2022, *n* = 8 with two subsamples per plot. For diurnal photosynthetic measurements, *n* = 3 with three subsamples per plot and significance was determined with ANOVA on the linear model and post hoc Dunnett's test against the control. Harvested tubers were analyzed according to the RCB linear model with ANOVA and post hoc Duncan testing. For tuber nutrient analysis in 2020 *n* = 6 with three subsamples per plot and in 2022 *n* = 8 with two subsamples per plot and significance determined by the linear model with ANOVA and post hoc Duncan tests. No block effects were observed in any measurements.

Leaf photorespiratory metabolites were analyzed from a growth chamber experiment where *n* = 8 plants per line and statistical significance determined with a one‐way ANOVA and post hoc Dunnett's test where a = 0.05. Statistical analysis was performed in R (version 4.2.1 https://www.R‐project.org/).

## 3 Results

### 3.1 Production and Selection of Potato Transformants

*Solanum tuberosum* cv. Desiree was genetically transformed by BTI *Agrobacterium tumefaciens* strain 3C5ZR with kanamycin selection according to Van Eck et al. (2007) at The Boyce Thompson Institute (Cornell University, NY, USA). In this study, we used two versions of alternative pathway constructs (Figure S1). The first, EC36030 (AP3), was transformed to overexpress plant malate synthase (cmMS) and *Chlamydomonas reinhardtii* glycolate dehydrogenase (CrGDH). The introduced genes divert flux from the native pathway to metabolize glycolate in the chloroplast (Figure 1). The second construct, EC36031 (AP3 + RNAi), introduced the same two genes with the addition of an RNA interference (RNAi) module to reduce the expression of the glycolate–glycerate transporter (PLGG1) to minimize an export of glycolate from the chloroplast into the peroxisome as in the native photorespiratory pathway (Figure 1). The transformed plantlets were screened with qRT‐PCR to confirm the expression of AP genes before choosing events for field testing. Three events from each construct with confirmed expression were carried forward for field testing.

### 3.2 Gene Expression Confirmed in Field‐Grown Plants

From preliminary field trials in 2019 using three events per construct, the best performing event from each construct was selected to enable greater repetition for robust statistical power in subsequent field testing. Best performing events were determined as those with the highest tuber mass per plant (AP3 line 36030–9, and AP3 + RNAi line 369031‐17) (Figure S2a). Gene expression of MS and GDH was confirmed with qPCR in both events, and reduced PLGG1 expression confirmed in AP3 + RNAi event 369031‐17 (Figure S2b–d).

**FIGURE 2:**
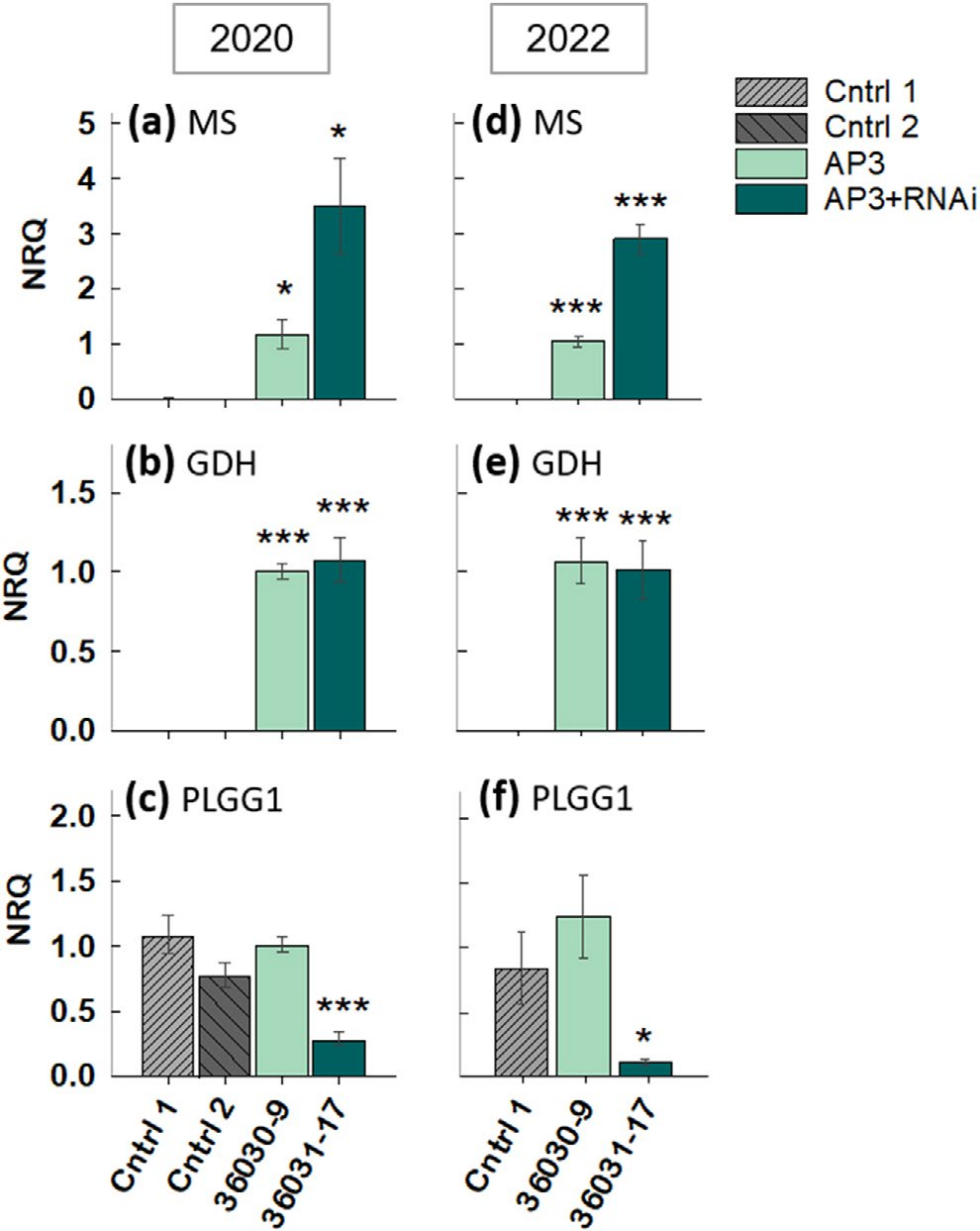
Increased RNA expression of introduced genes MS and GDH and decreased expression of PLGG1. qRT‐PCR analysis of introduced AP3 genes, MS (a, d) and GDH (b, e) and RNAi target gene PLGG1 (c, f), for plants grown in 2020 and 2022 field trials. Data are normalized to the AP3 event and expressed as normal relative quantity (NRQ) using reference genes Cyc and Tub. *n* = 6 in 2020, *n* = 8 in 2022. Asterisks denote significant differences in expression levels between transgenics and controls, using two tailed *t*‐tests in panels against combined control mean for panels a–c, and against control 1 in Panels d–f. a = 0.05 for all statistical tests. *p*‐values are listed in Table S6.

Transgene qRT‐PCR expression analysis for plants grown in 2020 and 2022 field trials showed MS and GDH expression for AP3 and AP3 + RNAi events (Figure 2a,b,d,e) and PLGG1 knocked down in the AP3 RNAi events (Figure 2c,f).

### 3.3 AP3 Pathway Increases Tuber Mass

**FIGURE 3:**
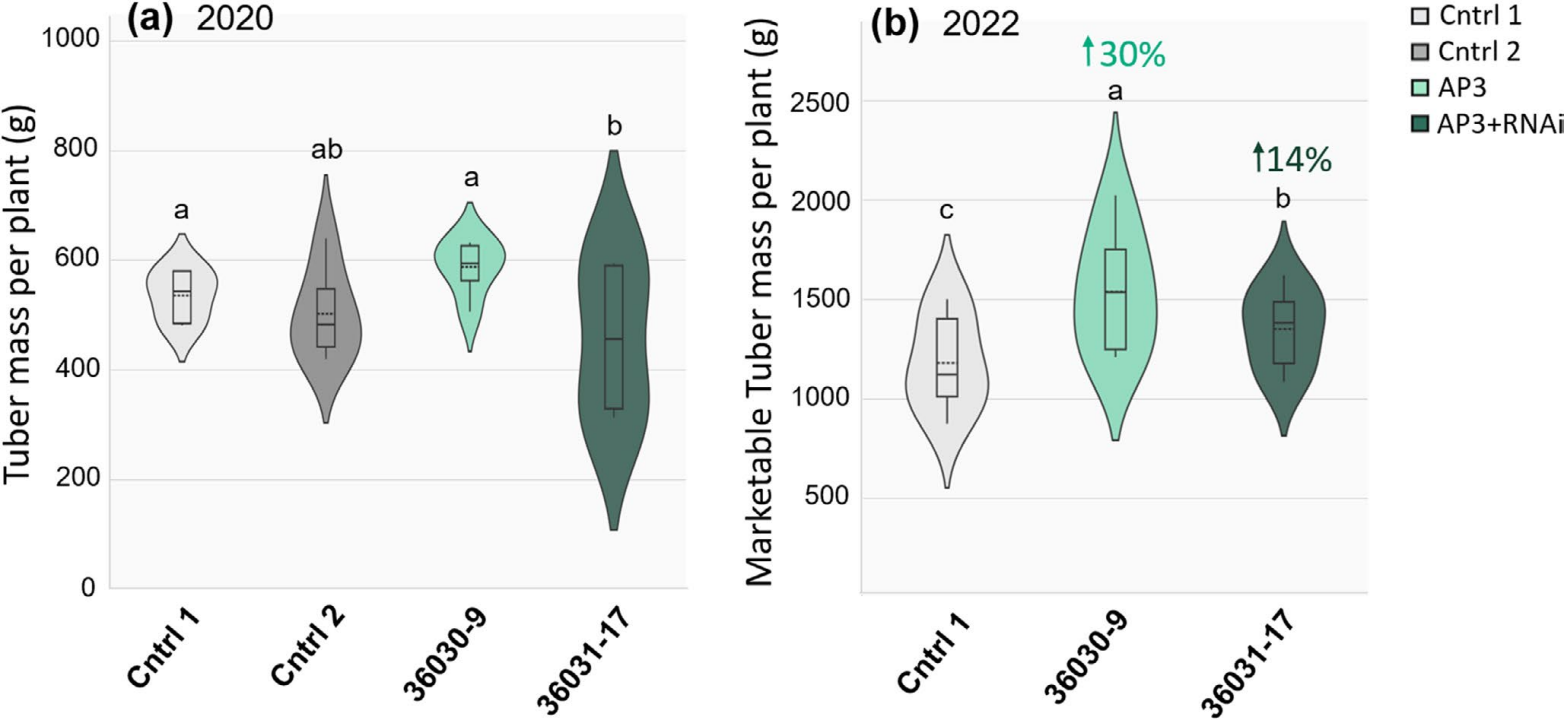
Increased tuber mass per plant in 2020 and 2022 field trials. Tuber mass per plant from 2020 (a) and 2022 (b) field trials, calculated as total tuber weight per plot divided by plants per plot for tubers > 2 g in 2020, and tubers > 5 g (marketable weight) in 2022. Solid vertical lines represent the median, dashed vertical lines represent the mean, black boxes indicate interquartile ranges, and vertical black lines represent the remaining distribution. In 2020, *n* = 6 plots with 10 subsamples per plot (a), *n* = 8 plots with 10 subsamples per plot (b). Letters indicate statistical significance based on linear model ANOVA, *y* = *μ* + Block + Line + *ε* with post hoc Duncan tests (a = 0.05) where for (A) 9.5% increase in 36030‐9 compared with Cntrl 1, *p* = 0.05 (B) 30% increase *in* 36030‐9 (*p* = 0.0006) and 14.2% increase 36031‐17 (*p* = 0.049) compared with Cntrl 1. Statistics for linear model ANOVA are listed in Table S7.

In the 2019 growing season, three independent AP3 events and three independent AP3 + RNAi events were tested against two untransformed controls in a randomized complete block (RCB) field experiment where *n* = 3 plots (Figure S3a). AP3 line 36030‐9 showed a 23% increase in tuber mass per plant compared with the best‐performing control (Cntrl 1) where *p* = 0.04. AP3 + RNAi line 36031–17 showed a 7.5% increase (*p* = 0.05) compared with Cntrl 2 (Figure S2a) warranting subsequent field testing of these best‐performing lines. In 2020, AP3 event 36030‐9 and AP3 + RNAi event 36031‐17 were grown in an RCB field experiment where *n* = 6 plots (Figure S3b) showing 9.5% increase in tuber mass per plant for AP3 line 36030‐9 (*p* = 0.05) compared with best‐performing control (Cntrl 1) (Figure 3a). In 2022, the consistently poorest performing control (Cntrl 2) was removed from the experiment allowing an RCB field test where *n* = 8 plots (Figure S4c), showing an increase in tuber mass per plant of 30% (AP3, 36030–9 where *p* = 0.0006) and 14% (AP3 + RNAi, 36031–17 where *p* = 0.049) compared with Cntrl 1 (Figure 3b). In 2020, there were no differences in average mass per tuber (Figure S4a), but in 2022, a 14% increase in mass per marketable tuber was apparent in the AP3 + RNAi line compared with the control (Figure S4b).

### 3.4 Diurnal Measurements Reveal Higher CO_2_ Assimilation Rates in AP3 Plants

**FIGURE 4:**
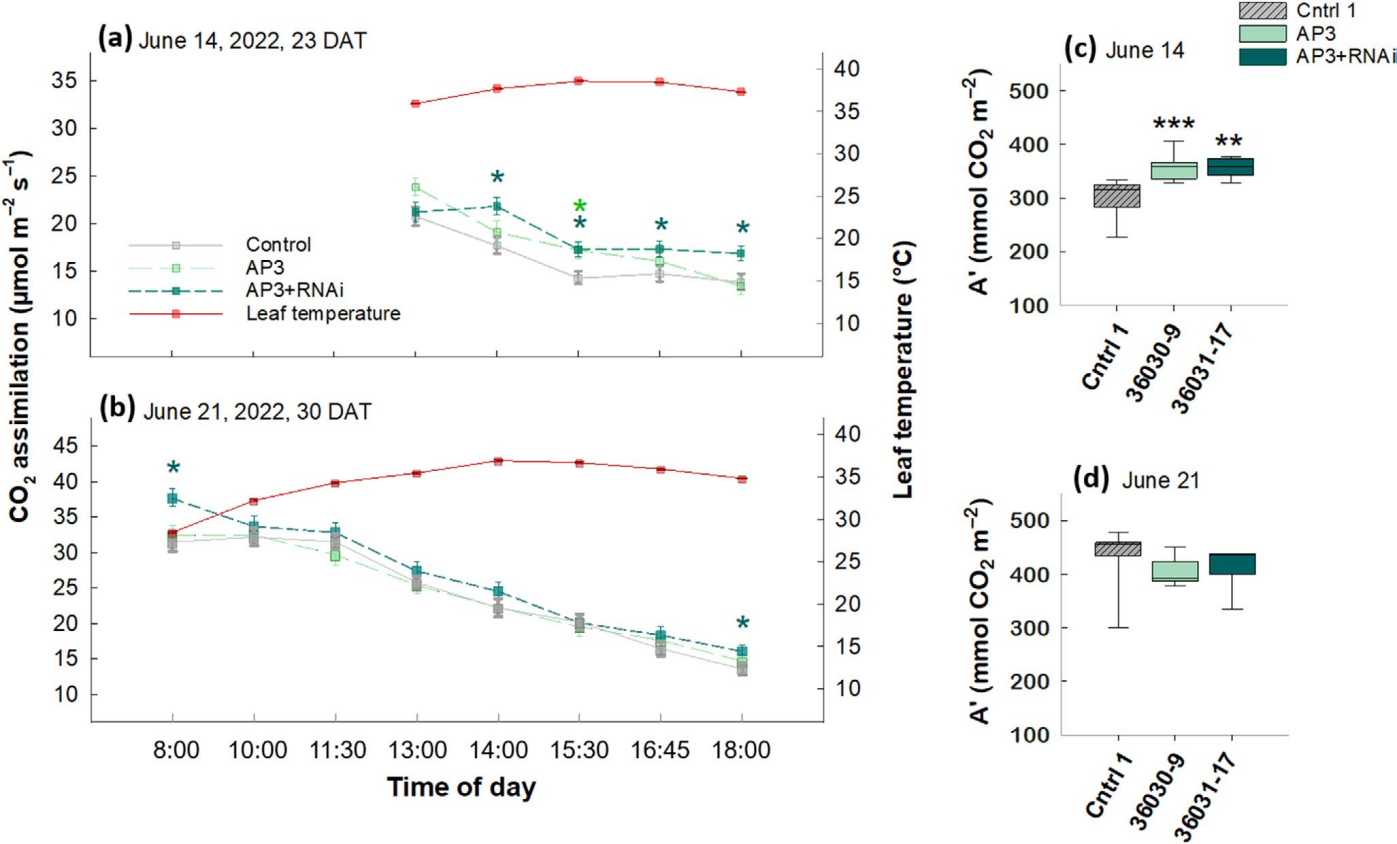
Increased diurnal CO_2_ assimilation in 2022. Diurnal course of CO_2_ assimilation measured on (a) June 14 (23 DAT) and (b) June 21 (30 DAT), 2022, collected with survey measurements of photosynthetic gas exchange. *n* = 8 plots with three subsamples per plot. Fixed carbon (A') from 13:00 to 18:00 h is calculated from area under the curve per block for June 14 (c) and June 21 (d). On June 14th, AP3 showed an 18% increase in fixed carbon and (AP3 + RNAi) a 19% increase compared with controls (c). Solid box‐plot lines indicate interquartile ranges and means, whisker caps denote the 90th and 10th percentiles. Asterisks in all panels show significance based on linear model ANOVA, *y* = *μ* + Block + Line + *ε* with post hoc Dunnett's tests (a = 0.05). Statistics including *p*‐values are shown in Table S8.

Given that no significant increases in photosynthetic capacities, leaf optical or diffusional properties (*V*_cmax_, *J*_max_, ɸPSII and ɸCO_2,_ g_m_ and leaf absorptance) were seen in the 2020 season (Figure S5), and our observation that AP3 + RNAi offers thermal protection to modified tobacco plants (Cavanagh et al. 2022), measurements of diurnal CO_2_ assimilation under heat stress became the focus for 2022. We captured two diurnal measurement sets in two of three observed natural heatwaves, recording higher rates of CO_2_ assimilation in the bypass plants over multiple time points (Figure 4a,b). At 23 DAT (June 14, 2022) during mid‐vegetative growth, plants expressing the AP3 pathway showed a 14% greater CO_2_ assimilation rate relative to the control at 13:00 and a 20% greater rate at 15:30 pm, and AP3 + RNAi plants had higher rates ranging from 17% to 23% between 14:00 and 18:00 h (Figure 4a; Table S2). Due to variable cloud cover, which violates the requirement for homogeneous light conditions for comparable survey measurements of CO_2_ assimilation, no measurements were made in the morning on the first diurnal measurement at 23 DAT (June 14, 2022). During diurnal measurements on June 21 at 30 DAT (Figure 4b), the AP3 + RNAi line showed 20% higher CO_2_ assimilation rates compared with the control at 8:00 and 18:00 only (Figure 4a; Table S2), with no significant differences observed for the AP3 line. The two measurement days represent not only differences in vegetative development but also differences in the thermal environment. Calculation of iWUE (intrinsic water use efficiency) as net photosynthetic assimilation/stomatal conductance (A_n_/g_s_) for both diurnal measurement days showed no difference between lines at any measured time point (Figure S8).

The total carbon fixed over the afternoon period (A'), integrated for each diurnal from 13:00 to 18:00 h, also reflects the differences between the measurement days, with AP3 lines fixing 18% (AP3) and 19% (AP3 + RNAi) more carbon than controls (Figure 4c), while no differences were observed between any genotypes on June 21 (Figure 4d). These differences are likely driven by temperature, as the relationship between leaf temperature and CO_2_ assimilation throughout the afternoon (i.e., 13:00–18:00 h) is negatively correlated in all lines on June 14 (Figure S6a), suggesting that plants were experiencing heat stress. On June 21, there is no relationship during afternoon hours (Figure S6c).

### 3.5 Photosynthetic Capacity Increases and CO_2_ Compensation Point Decreases in AP3 Plants During Tuber Bulking

**FIGURE 5:**
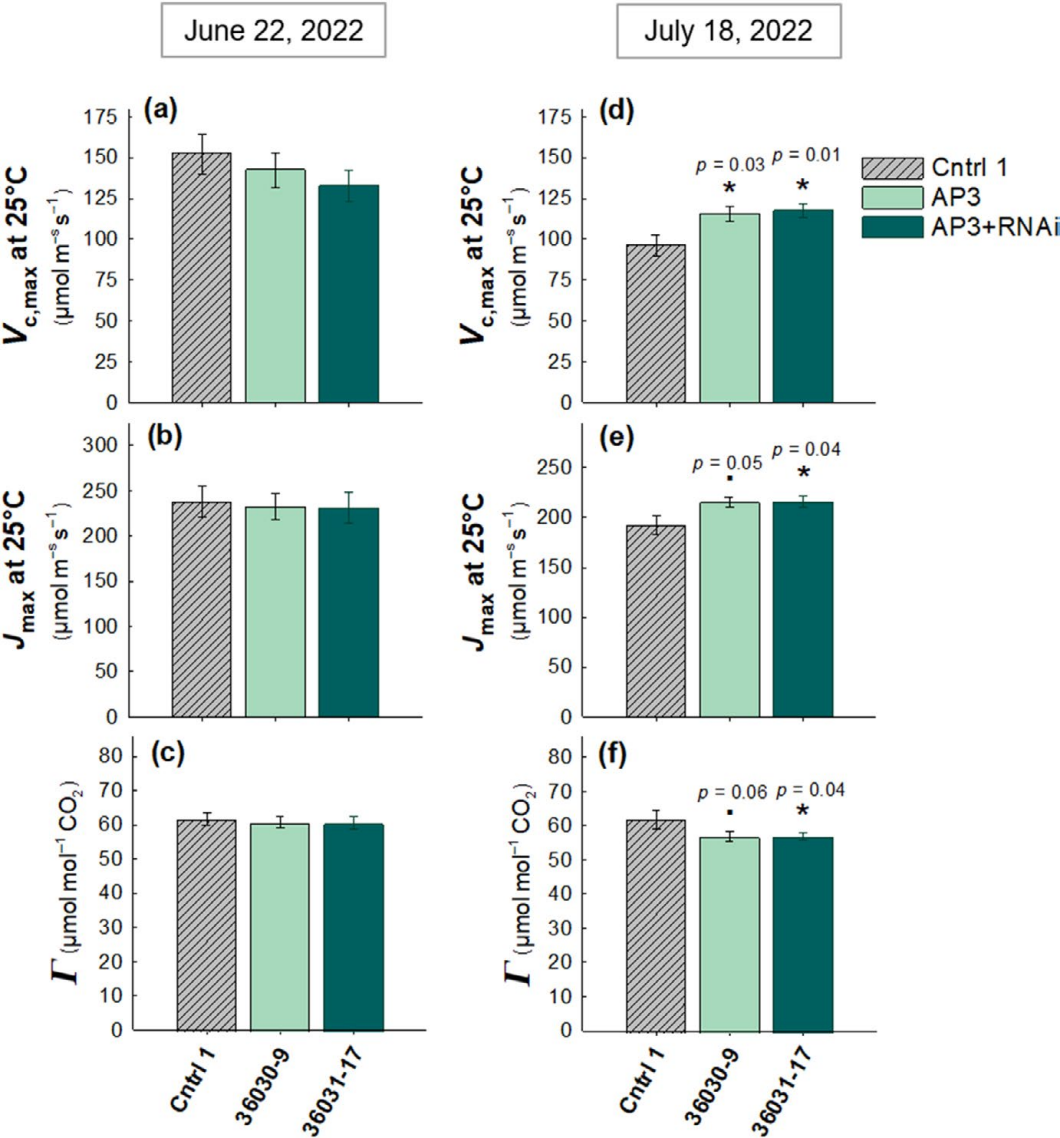
Increased photosynthetic capacity and reduced CO_2_ compensation point in late season measurements. *V*_cmax_ and *J*_max_ at 1800 μmols PAR m^−2^ s^−1^, and *Γ*, all modelled at 25°C from photosynthetic (*A*) vs. intercellular CO_2_ (*C*_*i*_) response curves measured on June 22, 2022 at 31 DAT (a–c) and July 18, 2022 at 57 DAT (d–f). On July 18th, *V*_cmax_ increased by 20% and 23% in AP3 and AP3 + RNAi, respectively (d) *J*_max_ increased by 12% and 13% compared with controls (e), and Γ decreased by 8% in both transgenic lines compared with controls (f). *n* = 8 plots with two subsamples per plot. All error bars show SEM. Asterisks indicate statistical significance based on linear model ANOVA, *y* = *μ* + Block + Line + *ε* with post hoc Dunnett's tests (a = 0.05). Statistics including p‐values are shown in Table S9.

Photosynthetic capacities measured on June 22 (31 DAT) during vegetative growth, showed no change in *V*_cmax_ or *J*_max_ (Figure 5a,b respectively). Later in the season on July 18 (57 DAT) during mid tuber bulking, photosynthetic capacity increased in the transgenic lines with a significant 20% and 23% increase in *V*_cmax_ in the AP3 and AP3 + RNAi lines, respectively, compared with the control (Figure 5d)_._ *J*_max_ increased by 12% (*p* = 0.05) in the AP3 line and by 13% in AP3 + RNAi line (Figure 5e). While no differences between lines were observed in the CO_2_ compensation point (Γ) on June 22 (Figure 5c), Γ was reduced in both AP3 lines by 8% compared with the control by July 18th (Figure 5f).

In 2022, no differences were observed in ɸCO_2_ or leaf absorptance values between any line (Figure S7).

### 3.6 Seasonal Temperature and Plant Growth Stages

**FIGURE 6:**
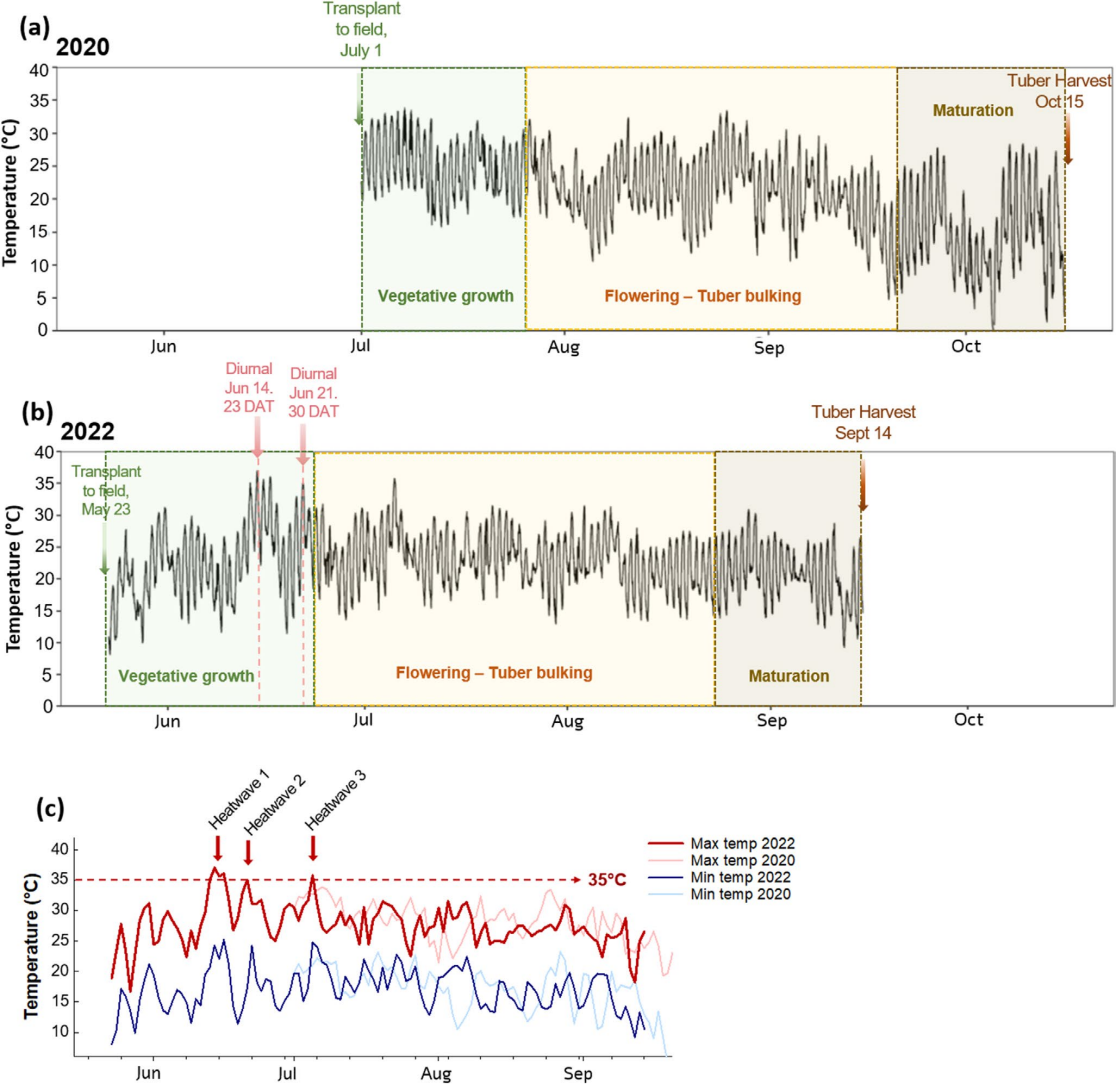
Seasonal growing season temperature. Season temperatures (°C) for 2020 (a) and 2022 (b) averaged for every 10 min from sowing date to harvest. Green arrows indicate sowing dates, brown arrows indicate harvest dates and red arrows indicate the date diurnal measurements of CO_2_ assimilation were measured in 2022, with potato growth stages for each season highlighted. (c) Overlays daily maximum and minimum temperatures for both 2020 and 2022, with periods of natural heatwave (> 35°C) highlighted.

In 2020, potato plantlets were propagated later than desired due to AHPIS permitting delays and field transplant was delayed until July 1st (Figure 6a). From transplant through vegetative growth, daily temperature ranges remained fairly consistent, with average daily maximum temperatures of 30.6°C ± 2°C and minimum temperatures of 19°C ± 2.1°C. Through flowering and tuber bulking temperature remained fairly consistent with an average maximum daily temperature of 27.2°C ± 3.2°C and minimum of 15.9°C ± 4°C. During maturation from the onset of leaf senescence through to tuber harvest, temperatures cooled with average highs of 22.7°C ± 4.9°C and lows of 7.4°C ± 4.1°C (Figure 6a). In 2022, potato plantlets were transplanted to the field in late spring on May 23rd (Figure 6b). From transplant through vegetative growth, plants experienced cooler temperatures at establishment but overall higher daily maximal temperatures during late vegetative growth when compared to vegetative growth in 2020. An unseasonal heatwave event occurred between June 13 and June 16, 2022, with temperatures reaching over 35°C for four consecutive days during vegetative growth, followed by a return to seasonal temperatures for 3 days before another 35°C day on June 22, 2022 (Figure 6b). Through flowering and tuber bulking 2022 temperatures were fairly consistent with average highs of 28°C ± 2.5°C and lows of 17.8°C ± 2.8°C. During maturation in 2022, temperatures cooled with average highs of 26.7°C ± 2.9°C and lows of 15.5°C ± 3.1°C (Figure 6b), with higher average temperatures during maturation than were experienced in 2020 due to earlier senescence and harvest. In 2020, daily maximum temperatures never reached above a maximum 33.8°C (July 7th) throughout the entire growing season, yet three early season heatwaves with maximum daily temperature above 35°C were observed in 2022 (Figure 6c).

### 3.7 No Penalty Observed in Tuber Nutritional Composition in Bypass Events With Biomass Increases

**FIGURE 7:**
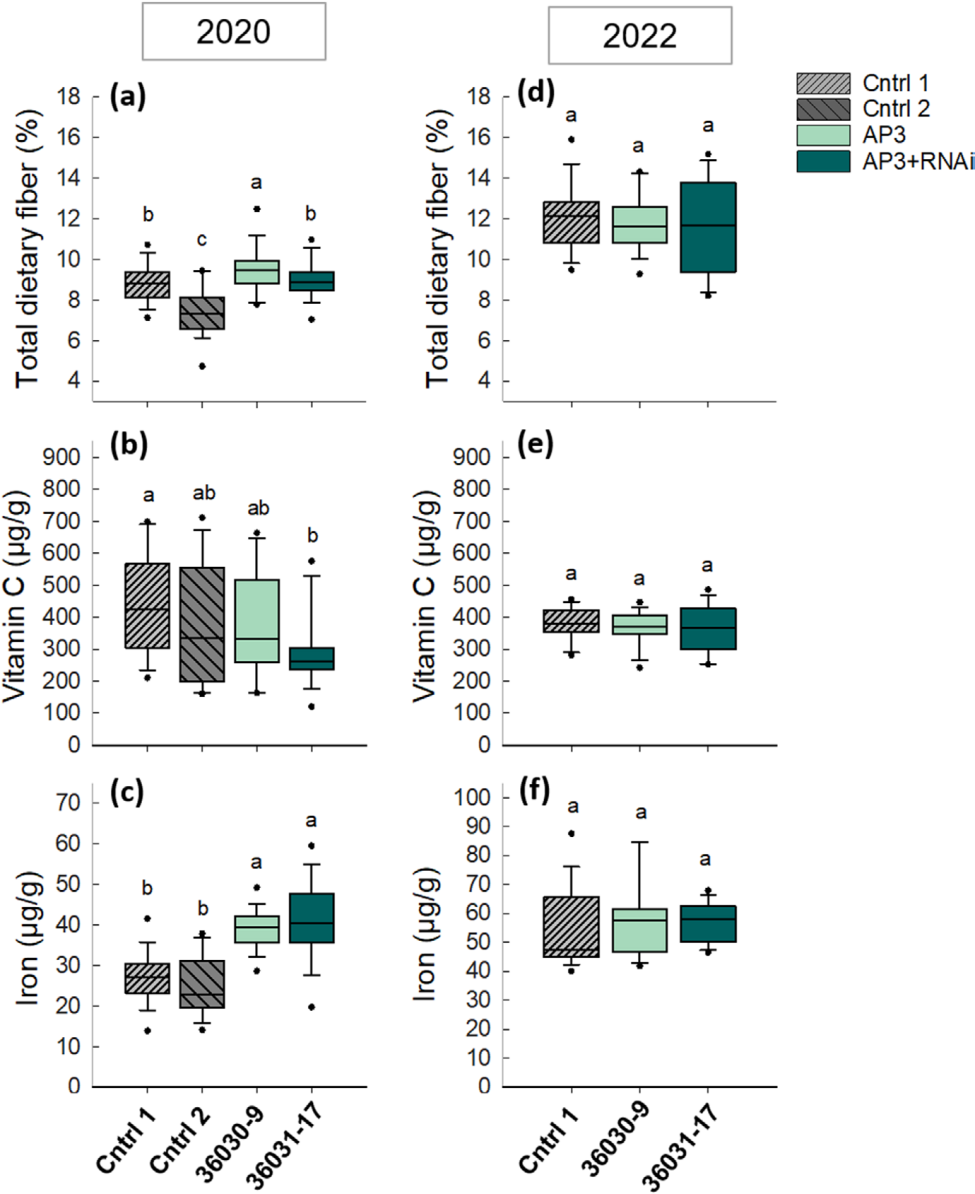
Nutritional composition of transgenic potato tubers. Tuber dietary fiber, vitamin C, and iron content in 2020 (a–c) and 2022 (d–f). Values are presented on a dry matter (DM) basis. In 2020, *n* = 6 with three subsamples per plot. In 2022, *n* = 8 with two subsamples per plot. Solid box‐plot lines indicate interquartile ranges and means, whisker caps denote the 90th and 10th percentiles and outliers are represented by dots. Letters indicate statistical significance based on linear model ANOVA, *y* = *μ* + Block + Line + *ε* with post hoc Duncan tests where a = 0.05. The full suite of nutrient content assays in 2020 with no significant differences are shown in Table S3.

In 2020, while no differences between transgenic and control tubers were observed for starch, protein, amino acids, free sugars, calcium, and potassium (Table S3), an 8% increase in total dietary fiber (TDF) was apparent in the AP3 line (Figure 7b), decreased Vitamin C (27%) in the AP3 + RNAi line (Figure 7b), and increased iron in both the AP3 (44%) and AP3 + RNAi line (52%) (Figure 7c). All percentage change values are compared with the best‐performing control, Cntrl1. In 2022, no changes in nutritional composition were observed for any assay (Figure 7d–f).

### 3.8 AP3 Alters Leaf Photorespiratory Metabolite Pools

**FIGURE 8:**
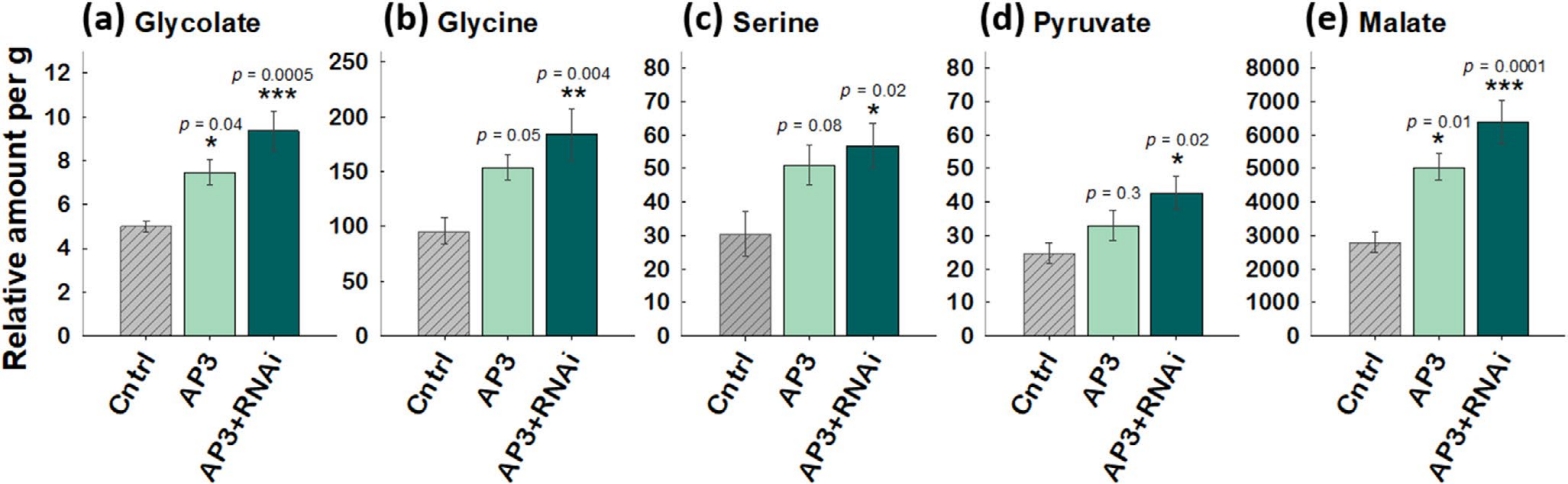
Leaf photorespiratory metabolite analysis. Relative amount of metabolites from ~100 mg of fresh, flash frozen potato leaf tissue from samples collected from plants grown in growth chamber conditions on day 4 of an imposed heatwave. Metabolites were analyzed by gas chromatograph mass spectrometry and metabolite concentrations reported per gram fresh weight. Error bars indicate SEM, *n* = 8 leaf samples. Asterisks indicate statistical significance from one‐way ANOVA and post hoc Dunnett's tests where a = 0.05, with *p* values indicated.

In a growth chamber experiment where potato plants experienced simulated heatwaves mimicking the 2022 early field season growth patterns, mass spectrometric analysis showed an increased accumulation of glycolate, glycine, serine, pyruvate, malate (Figure 8a–e respectively), in leaves of AP3 lines compared with the control, showing altered photorespiratory metabolism. Glyoxylate levels were undectable in any line at analysis resolution.

## 4 Discussion

In this study, we have shown that an alternative photorespiratory pathway (AP3) increased tuber mass in a globally important agricultural food crop over two growing seasons. We built on previous work that showed AP3 improved growth and performance of field‐grown model crop tobacco under seasonal conditions (South et al. 2019) and in field conditions under elevated heat stress (Cavanagh et al. 2022). We engineered the AP3 pathway into potato cv. Desiree, hypothesizing that the introduction of genes to metabolize glycolate in the chloroplast would cause increased leaf photosynthetic rates by reducing the export of glycolate into the costly native photorespiratory pathway and increase thermotolerance under heat stress, ultimately resulting in potato tuber yield increases.

We demonstrated 9%–30% increases in tuber biomass in replicated single location field experiments across 2 years and confirmed that this yield benefit does not come at the expense of tuber quality (Figure 7). In 2020, we saw a 9.5% tuber mass per plant increase in AP3 compared with controls (Figure 3a), but increases in photosynthetic capacity were not detectable (Figure S5). In 2022, we observed a 30% and 14.2% increase in tuber mass per plant in AP3 and AP3 RNAi lines, respectively, compared with controls (Figure 3b), and observed temporal increases in both photosynthetic rates (Figure 4) and photosynthetic capacity (Figure 5) after early season heatwaves (Figure 6), which highlights the interplay between photosynthetic metabolism, development, and acclimation to heatwaves with daily max temperature greater than 35°C. The reduction in CO_2_ compensation point in AP3 lines during tuber bulking (Figure 5f) and the altered pool sizes of photorespiratory metabolites in AP3 compared with controls (Figure 8) indicate the photosynthetic increases and tuber biomass gains are a result of a functioning AP3 pathway diverting flux from native photorespiration, accelerated by heat stress during early growth.

### 4.1 AP3 Function Increases Photosynthetic Rates and Capacity

The AP3 pathways are predicted to enhance photorespiratory CO_2_ refixation in the chloroplast, via the oxidation of glycolate to glyoxylate, which is converted to malate via MS, and subsequently decarboxylated, resulting in the release of two molecules of CO_2_ in the chloroplast (South et al. 2019; Aboelmy and Peterhansel 2014; Beezley, Gruber, and Frederick 1976; Figure 1). CrGDH is a mitochondrial localized enzyme in *Chlamydomonas* where it uses ubiquinone as an electron acceptor. In plant chloroplasts, CrGDH localizes to the thylakoid membrane (South et al. 2019) potentially allowing it access to plastoquinone as an electron receptor, which would capture a significant amount of redox energy contributing to the energetic advantage over the native pathway where this redox energy is released as heat when peroxisomal glycolate oxidase reduces oxygen; thus, theoretical photosynthetic performance would be improved. Indeed, previous studies have shown AP3 tobacco plants to have altered photorespiratory metabolite profiles and improved photosynthetic performance compared with controls (Cavanagh et al. 2022; South et al. 2019), including enhanced carboxylation capacity (*V*_cmax_), quantum efficiency (ɸCO_2_), and quantum yield of photosystem II (ɸPSII) under photorespiratory stress. Similarly, in these potato studies, CO_2_ compensation points have been shown to reduce in AP3 lines compared with controls due to increased release of CO_2_ in the chloroplast. However, the photosynthetic benefit was not consistent in all environments.

In the preliminary study of AP3‐transformed tobacco from South et al. (2019), maximum carboxylation rates of Rubisco (*V*_cmax_) were shown to increase and CO_2_ compensation point reduced as hypothesized due to the increased availability of CO_2_ as a substrate for Rubisco at the site of carboxylation (South et al. 2019). However, Cavanagh et al. (2022), while observing lower CO_2_ compensation points at leaf temperatures over 40°, observed no increases in *V*_cmax_ in AP3 + RNAi lines, neither in ambient temperature nor under imposed heat stress (ranging from 15°C to 45°C) (Cavanagh et al. 2022) using tobacco plants with the same AP3 + RNAi construct design used in South et al. (2019). Instead, net CO_2_ assimilation measured over the diurnal period was shown to increase in transgenic lines compared to control plants in both studies, and this benefit is further enhanced under heat stress (Cavanagh et al. 2022), indicating that it is likely that cumulative diurnal increases rather than increased *V*_cmax_, drove the observed increases in AP3‐tobacco biomass compared with controls.

In 2022, we observed enhancements in photosynthetic capacity and reduced CO_2_ compensation points in transformed plants during tuber bulking stages (Figure 5d–f), but no differences in photosynthetic capacity were observed during early season vegetative growth (Figure 5a–c). The reduction of CO_2_ compensation point in AP3 lines during tuber bulking corresponds with the increases in photosynthetic capacity during this growth stage, supporting the notion that AP3 plants are better able to assimilate more carbon at low CO_2_ concentrations after heat stress. A similar heat stress protection is seen by Timm et al. (2019) in *Arabidopsis thaliana* photorespiratory mutants with 2‐PG overexpressed, where faster photorespiratory metabolism reduces *Γ* under heat stress. The lack of variation between lines in iWUE (Figure S8) indicates that AP3 benefits are indeed a result of altered photorespiratory pathways rather than physiological changes to stomatal opening.

Furthermore, given that limited tuber sink is known to reduce photosynthetic efficiency via carbohydrate accumulation in the leaves of potato plants (Basu et al. 1999), the developing tuber likely represents a strong photosynthate sink, which explains the temporal observations of increased *V*_cmax_ and *J*_max_ during tuber bulking (Figure 5d,e). These results highlight the importance of increasing crop sink capacity alongside improved crop photosynthetic performance to ensure increased source capacity translates to greater crop yields, particularly in grain crops (Paul, et al. 2020; Sonnewald and Fernie 2018). AP3 pathways may offer a greater advantage to tuber crops over grain crops considering that previous work in grain crops has concluded the yield loss driven by heat stress is a combination of the inhibition of photosynthesis and effects of high temperature directly on reproductive processes (Ferguson et al. 2021; Siebers et al. 2017; Templ and Calanca 2020). Because potato tuber yield does not depend on temperature‐sensitive reproduction (Ordoñez, Orrillo, and Bonierbale 2017), it is likely that the observed tuber mass increases in our study are due almost entirely to AP3 mitigation of impacts of temperature on photosynthetic physiology.

While the observance of increased metabolite pools in AP3 leaves under heat stress suggests that photorespiratory flux is altered by AP3 genes, targeted mechanistic assessment of metabolomic fluxes under different temperature regimes and growth stages as in Timm et al. (2021) would offer greater insight into AP3 function, given that single point in time metabolite analyses does not provide information about metabolic flux (Allen 2016; Koley et al. 2024).

### 4.2 AP3 Improves Temperature Acclimation and Thermotolerance

Given the delay of the field transplant in 2020, plantlets experienced very different early growth conditions between the 2 years. Vegetative potato plant growth thrives in cool conditions best supported in the air temperature range of 20°C–25°C (Fleisher et al. 2006; Kirk and Marshall 1992)^,^ while the optimal range for tuberization is even lower between 15°C and 20°C (Rykaczewska 2015; Van Dam, Kooman, and Struik 1996). High temperatures (28°C) during vegetative growth and early tuber formation have been shown to reduce stolon number and potential tuber sites in cv. Desiree over fivefold compared to optimal 18°C (Struik, Geertsema, and Custers 1989) and reduce tuber mass 10‐fold compared with control plants (Tang et al. 2018). In our 2020 field testing, plantlet establishment occurred in temperatures above the ideal 20°C–25°C and temperatures remained consistently above ideal for tuber development and maturation through the mid tuber bulking phase (Figure 6a). This, along with late field transplant, is likely contributed to the overall lower tuber yields in both AP3 lines and controls in 2020 compared with 2022 (Figure 3).

In 2022, an earlier field transplant facilitated optimal early season cooler temperatures during establishment and early vegetative growth, allowing optimal stolon formation and tuber initiation. In addition, plants experienced significant heat shock 3 weeks after transplant from June 13 to 16 (Figure 6b,c). Given the past observations of increased thermotolerance resulting from AP3 pathways, this heat shock likely enabled the significantly higher diurnal CO_2_ assimilation observed in AP3 and AP3 + RNAi plants compared with the control measured on June 14th (Daily high of > 37°C; Figure 4a,c). However, when measured 7 days later, no significant differences in the rate of carbon gain compared to controls were detectable (Figure 4d). While the second round of diurnal measurements of CO_2_ assimilation in 2022 was also carried out under unseasonably high temperatures on June 21 (daily high of 35°C), the leaves measured may have acclimated to a higher thermal environment. On June 21, measurements were made on leaves that had developed under a mean daily high temperature regime of 27°C (i.e., between Jun 3rd and 12th), as opposed to the leaves measured on June 14 which developed under a mean daily high temperature regime of 32.2°C (i.e., between June 11th and 20th). This distinction likely underpins the differential response, as pre‐existing leaves have less capacity to adjust or acclimate to a new growth temperature (Campbell et al. 2007; Way and Yamori 2014). In potato, temperature acclimation involves a shift of the thermal optimum (Yamori et al. 2010), and indeed, potato cultivars acclimated to high growth temperature maintain the rates of net photosynthesis similar to those grown at the thermal optimum (Wolf et al. 1990). Overall, our observations are consistent with the lack of an observed benefit to A' in AP3 + RNAi compared to WT controls in tobacco grown under cool temperatures (Cavanagh et al. 2022), supporting the hypothesis that modifications to photorespiration will offer photosynthetic thermal protection in high‐temperature conditions that promote high photorespiratory flux. It is likely that thermotolerance is increased in AP3 lines if introduced CrGDH maintains lower amounts of glycolate in the chloroplast of the AP3 plants. In this case, when glycolate production is high at high temperatures, AP3 is able to keep glycolate below the threshold that causes inhibition of the photosynthetic carbon reduction cycle.

While AP3 confers thermal protection at temperatures over 35°C in tobacco (Cavanagh et al. 2022), and our study suggests that a glycolate metabolic pathway enhances tuber mass in potato when heatwaves above 35°C occur during vegetative growth (Figure 6c), more species‐specific investigation is required into the critical temperature optimum for bypass benefits in potato. Furthermore, while our results suggest that mid/late vegetative growth high temperature stress is mitigated by AP3 pathways, more work is required to determine at which critical seasonal time points AP3 may have the best advantage.

### 4.3 Tuber Nutritional Content Is Uncomprimised by AP3

To determine if potato tubers from plants with altered photorespiratory metabolism displayed key differences in overall protein content or nutritional quality, in 2020, we examined a suite of tuber nutrients (Table S3) observing a large increase in tuber iron content in transformed plants and small differences in vitamin C and total dietary fiber (Figure 7a–c). A repeat of these three assays in 2022 showed no differences in nutritional content (Figure 7d–f).

Tuber protein content did not vary between either of the transgenic lines and controls (Table S3), though potatoes are low in protein in general. However, potatoes are an important dietary source of vitamin C and vitamin B6 and can also supply micronutrients and dietary fiber, with global potato consumption reaching ~35 kg/capita/year in 2013 (Wijesinha‐Bettoni and Mouillé 2019). Thus, any attempt to increase tuber yield via biotechnology must also consider the potential impact on nutritional content. AP3 genes caused no decline in tuber nutrient content in 2020 or 2022 (Figure 7; Table S3), yet in 2020, the increase in tuber yield observed in the AP3 event (Figure 3a) was accompanied by a 48% increase in tuber iron content (Figure 7c). Given that the large increases in iron content in both AP3 and AP3 + RNAi events (48% and 52%, respectively) in 2020 (Figure 7c) were not seen in 2022 (Figure 7f), the increases may have been due to a nutrient deficit or triggered due to insufficient time for iron accumulation due to a shifted growing season. Studies have shown that yield increases from elevated CO_2_ in rice (Ziska et al. 1996), wheat (Rogers et al. 1996), and tobacco (Geiger et al. 1999) were limited in correlation with limited nutrient supply, yet elevated CO_2_ improved plant iron content in tomato roots and shoots under iron‐limited conditions via increased ferric chelate reductase activity, increased root proton secretion for increased iron solubility, and the expression of *FER*, *FRO1*, and *IRT*, genes involved in iron uptake (Jin et al. 2009). It follows that the hypothesized increased chloroplastic CO_2_ availability stimulated by the AP3 pathway, in conjunction with potentially limited iron supply, may have caused a similar alleviation of iron deficiency induced in potato tubers. Increased CO_2_ assimilation has also been shown to increase sucrose accumulation (Bishop et al. 2018; Leakey et al. 2009; Stitt et al. 1988), in turn stimulating auxin‐signaled iron deficiency responses via FIT‐mediated transcriptional regulation of *iron uptake genes* in Arabidopsis (Lin et al. 2016). Given the demonstrated increase in CO_2_ assimilation and photosynthetic capacity in AP3 potatoes in this study, sucrose accumulation in AP3 potato plants may have increased potentially triggering a similar mechanism under iron deficiency, supported by increased tuber sucrose contents observed in transgenic lines compared with controls in 2020 (ANOVA *p* = 0.1) and 2022 (ANOVA *p* = 0.3) (Table S3). In our study, soil cores were not taken during 2020 field testing, but soil analysis of 2022 cores showed no iron deficiency (Table S4).

Although focused experiments aimed at understanding the relationship between iron uptake and photorespiratory mechanisms fall outside the scope of this study, investigation of the role of AP3 biochemistry in nutrient‐deficit soils may be important to explore expansion of potato growth into semi‐arid calcareous soils and to support potato iron biofortification research to address anemia in developing countries (Connorton and Balk 2019; Singh et al. 2022).

## 5 Conclusions

The increases in photosynthetic capacity and tuber mass in AP3 potato after early season heatwaves in this study present the AP3 pathway as a promising avenue for yield increases in the face of a warming planet. Considering the maintenance of tuber quality, our work anticipates that an alternative glycolate photorespiratory bypass may future proof crops, and root storage crops in particular, against not only increased global mean temperatures but also the likely increased intensity and duration of heatwave events forecast to result from global warming.

## Author Contributions

**Katherine Meacham‐Hensold:** conceptualization, data curation, formal analysis, investigation, methodology, writing – original draft. **Amanda P. Cavanagh:** conceptualization, data curation, formal analysis, investigation, methodology, writing – review and editing. **Peyton Sorensen:** data curation, formal analysis, investigation, writing – review and editing. **Jessica Fowler:** data curation, formal analysis, investigation. **Ryan Boyd:** data curation, formal analysis, investigation, review and editing. **Paul F. South:** conceptualization, data curation, formal analysis, investigation, writing – review and editing. **Jooyeon Jeong:** data curation, formal analysis, methodology, writing – review and editing. **Steven Burgess:** formal analysis, methodology, writing – review and editing. **Samantha Stutz:** data curation, formal analysis, writing – review and editing. **Ryan N. Dilger:** data curation, formal analysis, investigation, writing – review and editing. **Moonsub Lee:** data curation, investigation, writing – review and editing. **Nicholas Ferrari:** data curation, formal analysis, investigation, writing – review and editing. **Justin Larkin:** data curation, formal analysis, investigation, writing – review and editing. **Donald R. Ort:** conceptualization, funding acquisition, supervision, writing – review and editing.

## Conflicts of Interest

The authors declare no conflicts of interest.

## Supporting information


**Data S1** Supporting Information.

## Data Availability

Data from all manuscript figures are available at Illinois Public Data Repository (Meacham‐Hensold et al. 2024).
