# Supplementary material for: Shortcutting Photorespiration Protects Potato Photosynthesis and Tuber Yield Against Heatwave Stress
Source: Glob Chang Biol. 2024 Dec 4;30(12):e17595. doi: 10.1111/gcb.17595 (PMC11615717; doi:10.1111/gcb.17595)
Supplement: Supplementary file 1 — Data S1 Supporting Information. [file GCB-30-e17595-s001.docx]

**Supplementary Material**

**
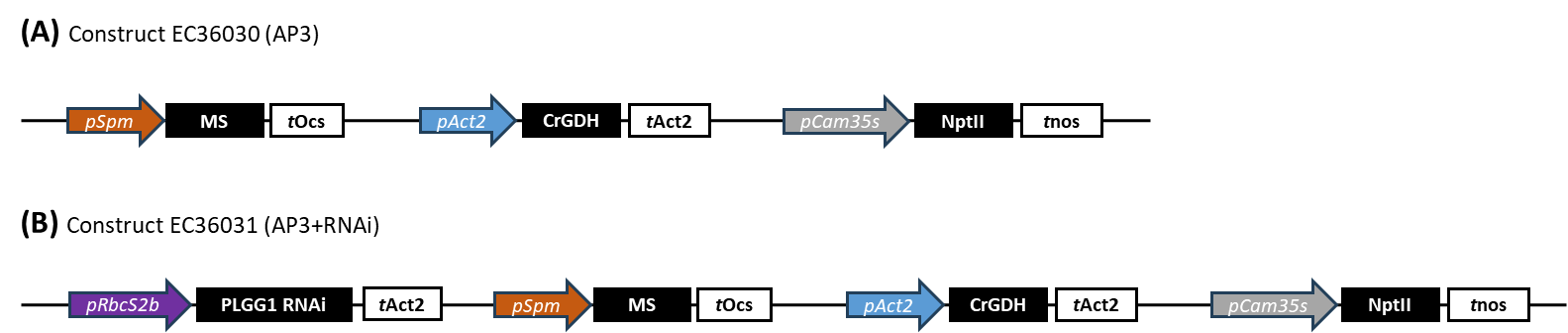
**

**Supplementary Fig. 1. Plasmid schematic of AP3 constructs transformed into *Solanum tuberosum* cv. Desiree*.***

AP3 construct EC36030 (A) introduces 2 functional genes, glycolate dehydrogenase (CrGDH) from *Chlamydomonas* *reinhardtii* and malate synthase (cmMS) from *Cucurbita maxima*, and a kanamycine resistance screenable marker neomycin phosphotransferase II (NPtII) from *Escherichia coli*. CrGDH is driven by the Actin 2 promoter (*pAct2*) from *Arabidopsis thaliana* and terminated by the Actin 2 terminator (*tAct2*) from *A. thaliana*. MS is driven by the maize suppressor-mutator transpossible element promoter (*pSpm*) and terminated by octopine synthase terminator (*tOcs*) from *Agrobacterium tumefaciens*. NptII is driven by the Cam35S promoter (*pCam25s*) from Cauliflower Mosaic Virus and terminated by nopaline synthase terminator (*tnos*) from *A. tumefaciens*. AP3+RNAi construct EC36031 (B) maintains the same design as construct EC36030 with the addition of a long hairpin RNAi module which targets the plastidic glycolate glycerate transporter PLGG1 from *Solanum tuberosum*. RNAi expression is driven by the Rubisco small subunit from *A. thaliana* (*RbcS2b*) and terminated by the Actin 2 terminator (*tAct2*) from *A. thaliana*.

**Supplementary Table 1**: **qPCR Primers used**

| St_GDH_primerF | AAGTTTATCCCCAAGGAGCG |
| --- | --- |
| St_GDH_primerR | CACCTTCACTACCAGCTTCG |
| St_Cyc_primerF | CTCTTCGCCGATACCACTCC |
| St_Cyc_primerR | TCACACGGTGGAAGGTTGAG |
| St_Tub_primerF | GGGAATAACTGGGCGAAAGGT |
| St_Tub_primerR | CCTCCACCAAGTGAGTGACAA |
| St_MS_primerF | GGGAATCTGAGTGGACATGTG |
| St_MS_primerR | CCAGAATTGAGTGCGTTGATG |
| St_PLGG1_primerF | GTCGCACTAGCTCTCAGCAT |
| St_PLGG1_primerR | ATTTGCTCCCACCAGACCAG |
| L25 RT F | CCCCTCACCACAGAGTCTGC |
| L25 RT R | AAGGGTGTTGTTGTCCTCAATCTT’ |


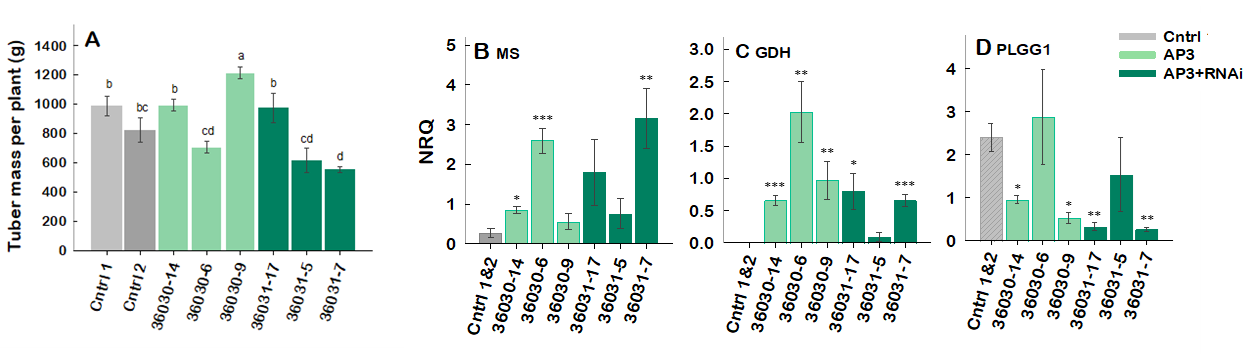


**Supplementary Fig. 2. Tuber mass per plant and gene expression in 2019**

(A) Tuber mass per plant calculated as total tuber weight per plot divided by plants per plot for tubers >2g. Error bars are SEM, n=3 plots. Letters indicate statistical significance based on linear model ANOVA, y = µ + Block + Line + ε with post hoc Duncan test. P = 0.04 for a 23% increase in tuber mass in AP3 line 36030-9 compared with Cntrl 1 and P=0.05 for a 7.5 % increase in AP3+RNAi line 36031-17 compared with Cntrl 2. Panels B,C and D present qRT-PCR analysis of introduced AP3 genes, MS (B) and GDH (C) and RNAi target gene PLGG1 (D), for plants grown in 2020 and 2022 field trials. Data are normalized to the AP3 event and expressed as normal relative quantity (NRQ) using reference genes StEif1A and L25. Asterisks denote significant differences in expression levels between transgenics and controls, using two tailed T-Tests where $a$=0.05 and n=3.

**
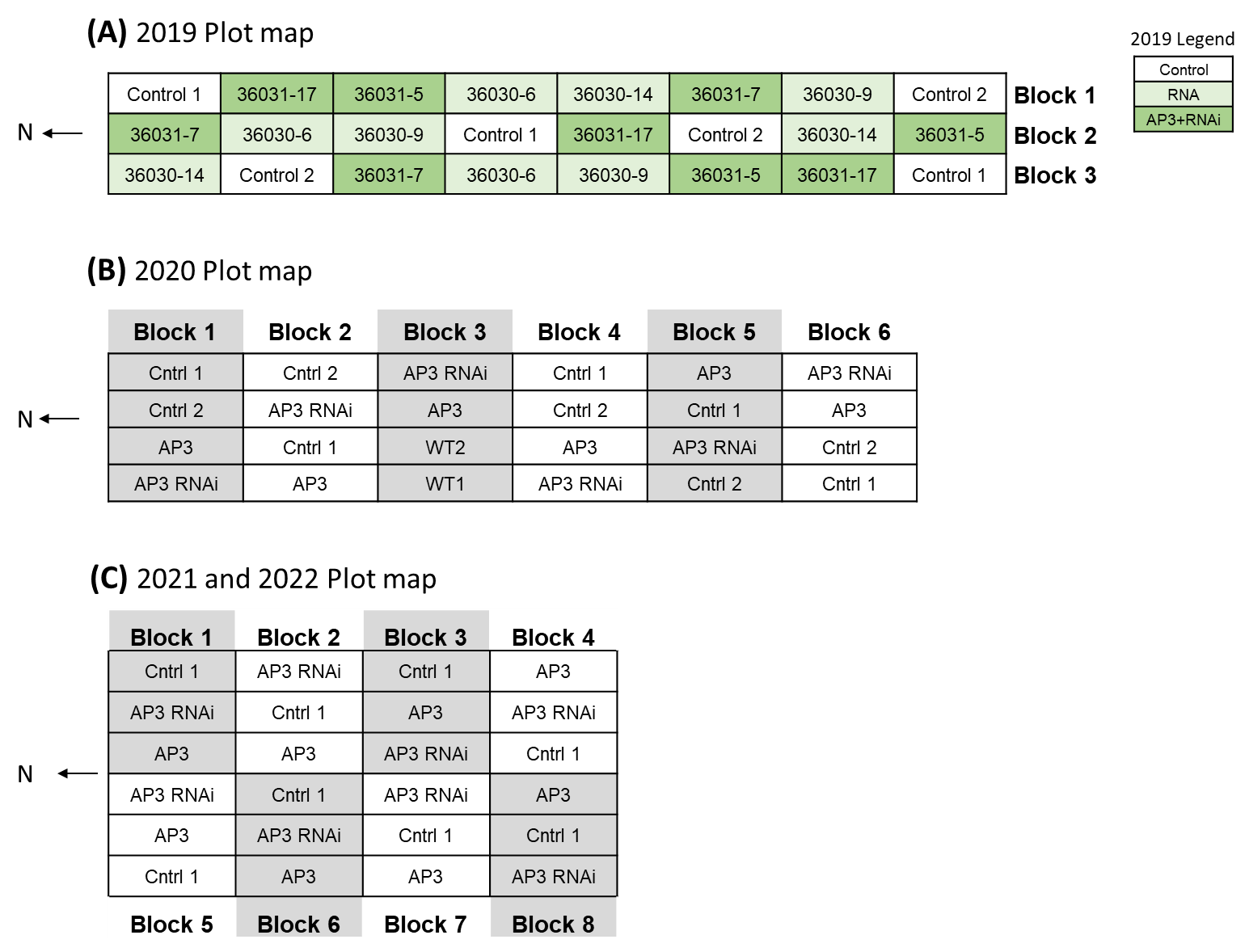
**

**Supplementary Fig. 3 Plot design for field trials in 2019, 2020, 2021 and 2022**

In 2019 field design consisted of 3 complete randomized blocks with 8 plots per block with 10 plants per block in a single row. Plants were spaced 12” apart with 20” between plots in a row, and 36” between rows. In 2020 (B), 6 complete randomized blocks were sown in 4 plots per block with 10 plants per block. In 2021 (flooded-out) and 2022 (C) 8 complete randomized blocks were sown with 3 plots per block. In all years plot density and spacing following the same protocol as in 2019.

**
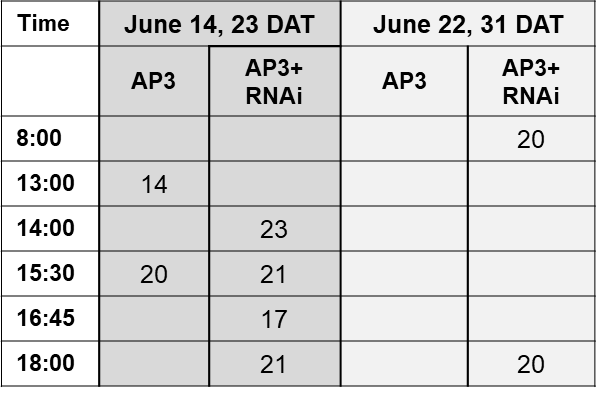
 Supplementary Table 2 – Percentage increase in CO_2_ assimilaton rates compared with control from main text Fig 4A&B**

**
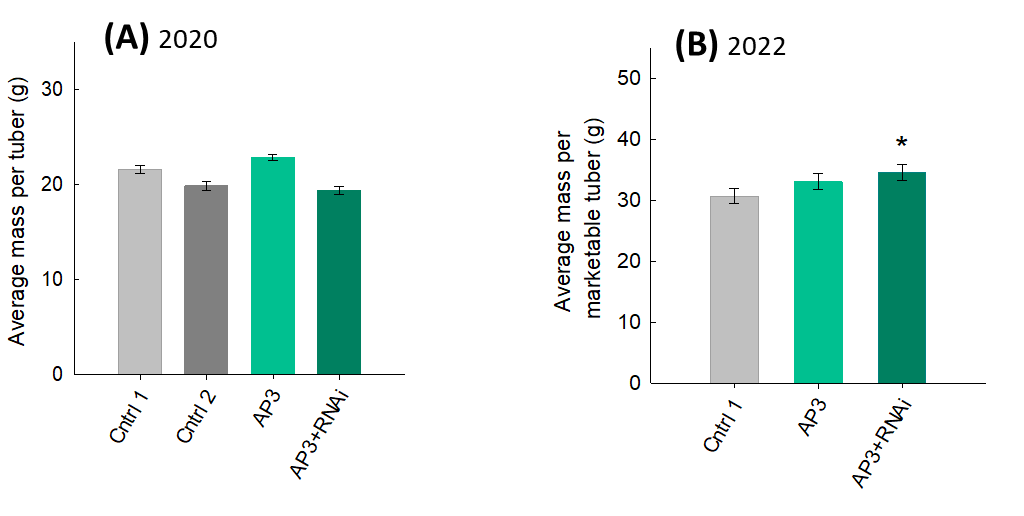
**

**Supplementary Fig 4. Average mass per tuber in 2020 and 2022 field trials.**

Average mass per tuber from 2020 (A) and average marketable mass per tuber 2022 (B) field trials, calculated as total tuber mass per plant divided by the amount of plants per plot. Error bars are SEM, n=6 (B), n=8 (C). Asterisk indicates statistical significance based on linear model ANOVA, y = µ + Block + Line + ε with post hoc Duncan tests where a=0.05, showing a 14% increase in average tuber mass in AP3+RNAi line 36031-17 compared with Cntrl 1 in 2022 (B).

 **Supplementary Fig. 5 Photosynthetic parameters 2020.**

V_cmax_ (A) and J_max_ (B) at 1800 µmols PAR m^-2^ s^-1^, modelled at 25°C from photosynthetic (*A*) vs. intercellular CO_2_ (*C_i_*) response curves measured on August 5-6^th^, 2020. From light response curves on the same days ɸPSII (C) was calculated as the slope of the relationship between the quantum yield of PSII and absorbed irradiance below 100 µmol m^−2^ s^−1^ and ɸCO_2_ (D) as the relationship between CO_2_ assimilation and absorbed irradiance below 100 µmol m^−2^ s^−1^. Mesophyll conductance (g_m_) (E) was calculated using carbon isotope discrimination (Δ^13^C) coupled with a gas exchange system under 1800 μmols PAR m^-2^s^-1^, 25°C and 20.9 kPa [O_2_]. Leaf absorptance (F) was measured with with a handheld spectrometer on Aug 5^th^ 2020 where measured absorption = 1-(transmittance+reflectance). For all parameters error bars show SEM and statistical significance was found based on linear model ANOVA, y = µ + Block + Line + ε where $a$=0.05.

**
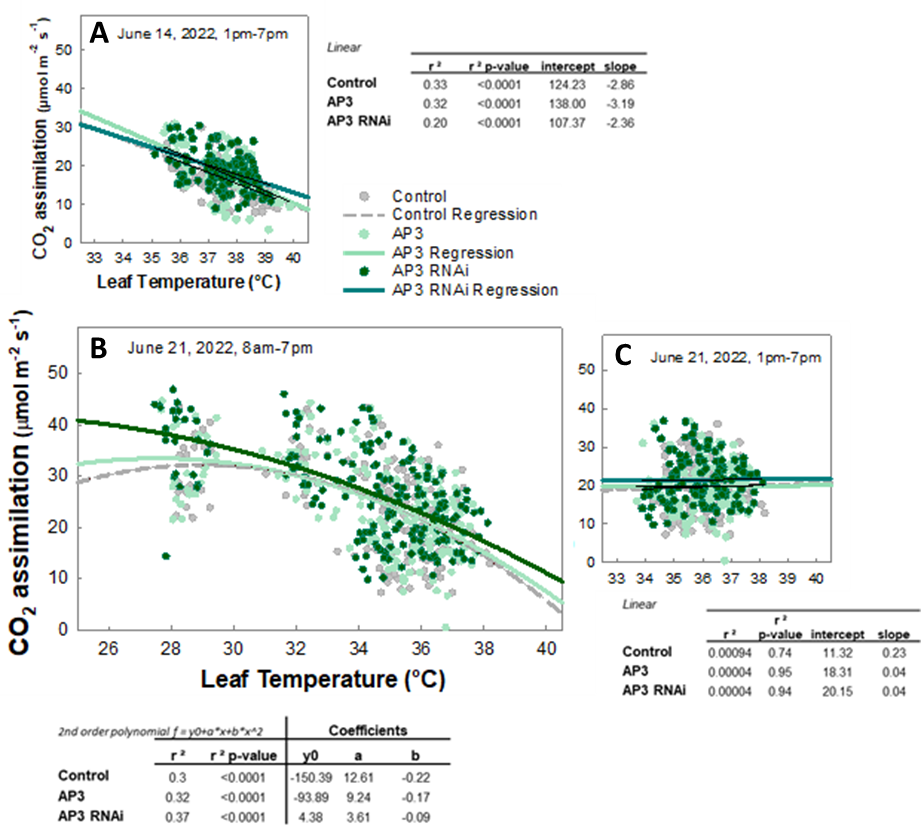
**

**Supplementary Fig 6. The relationship between leaf temperature and CO_2_ assimilation rates for diurnal measuremetns of photosynthesis**

The relationship between CO_2_ assimilation rates and leaf temperature for all diurnal measurements (13:00hrs-19:00 hrs) on June 14, 2022 with linear regression (A), and June 21, 2022 for all measurements (08:00 hrs-19:00 hrs) with 2^nd^ order polynomial regression (B). Call out depicts datapoints between 13:00 hrs-19:00 hrs for diurnal measurements made on June 21^st^, 2022 (C). Regression statistics and coefficients are summarized next to each figure panel A-C.


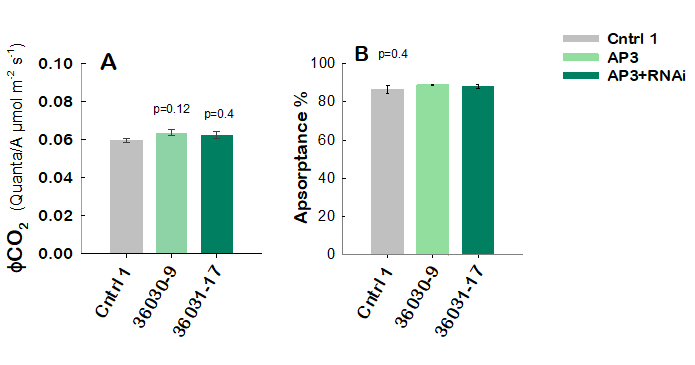


**Supplementary Fig. 7 ɸCO_2_ and leaf absorption in 2022**

Quantum yield of CO_2_ fixation (A), calculated as the initial slope of the relationship between absorbed PPFD (B) and CO_2_ assimilation from light response curves generated on Aug 30 2022 using LiCor 6800. n=8 plots with 2 subsamples per plot. Error bars show SEM. P values are derived from RCB linear model ANOVA, y = µ + Block + Line + ε with post hoc Dunnnett’s tests ($a$=0.05). (B) Leaf absorptance calculated from Jaz Spectrometer *(Ocean Optics)* measured on in-tact leaves in situ n=8 plots with 2 subsamples per plot. Error bars show SEM. P value is derived from single factor ANOVA.

**Supplementary Fig. 8. Diurnal Intrinsic Water Use Efficiency (iWUE)**

iWUE calculated over the diurnal on June 14, 2022 (A) and June 21, 2022 (B) as A_n_/g_s_ from survey measurements of photosynthetic gas exchange. n = 8 plots per line with 3 subsamples per plot (24 subsamples per line). All error bars show SEM. linear model ANOVA, y = µ + Block + Line + ε ($a$=0.05) showed no statistical differences between lines at any timepoint.


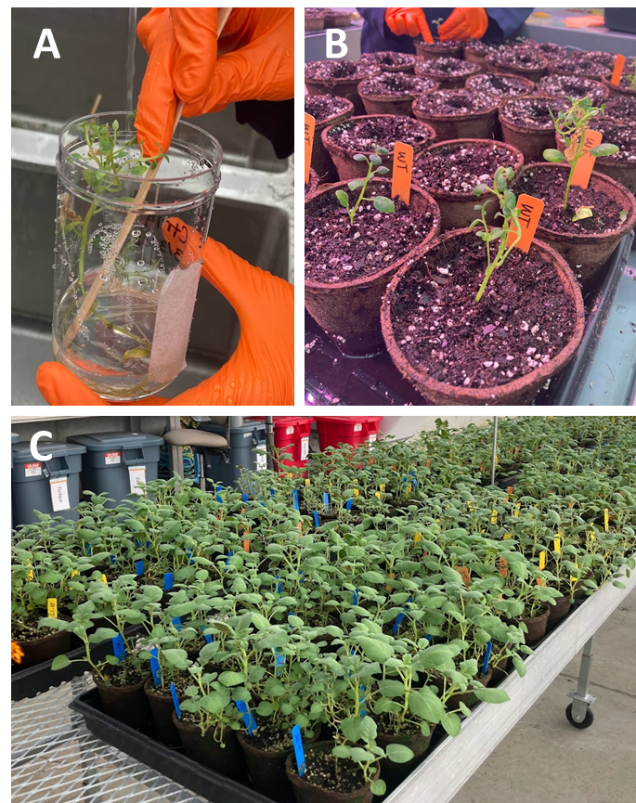


**Supplementary Figure 9. Plant establishment**

Tissue culture plantlets at eight weeks after establishment were extracted from tissue culture tubs (A) and transplanted into peat pots (B). After twelve days in growth chamber (Conviron) conditions 22°C/14hr day, 18°C/10 hr night at 700 μmol m^-2^ s^-1^ day light intensity and 50% RH, plants were moved into the greenhouse for five days prioir to field transplant for acclimation to diurnal solar conditions (26°C/14hr day, 20°C/10 hr night) (C).


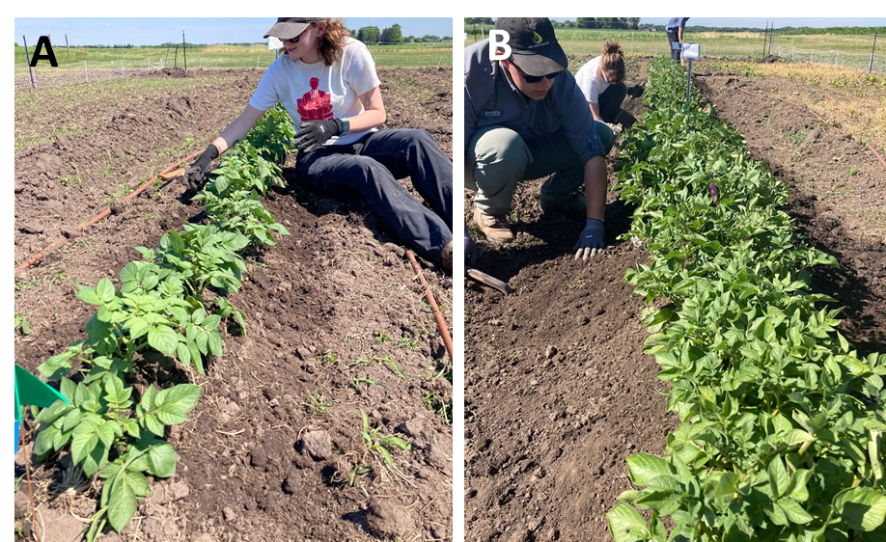


**Supplementary Figure 10. Hilling/Mounding**

Throughout growth, plants were hilled, in which soil is hand mounded to cover lower leaves about 20cm from ground level, to promote lateral tuber growth. In 2020 plants were hilled four times at 10, 25, 40 and 55 DAT. In 2020, plants were hilled at 10, 25 (A) and 40 (B) DAT

**
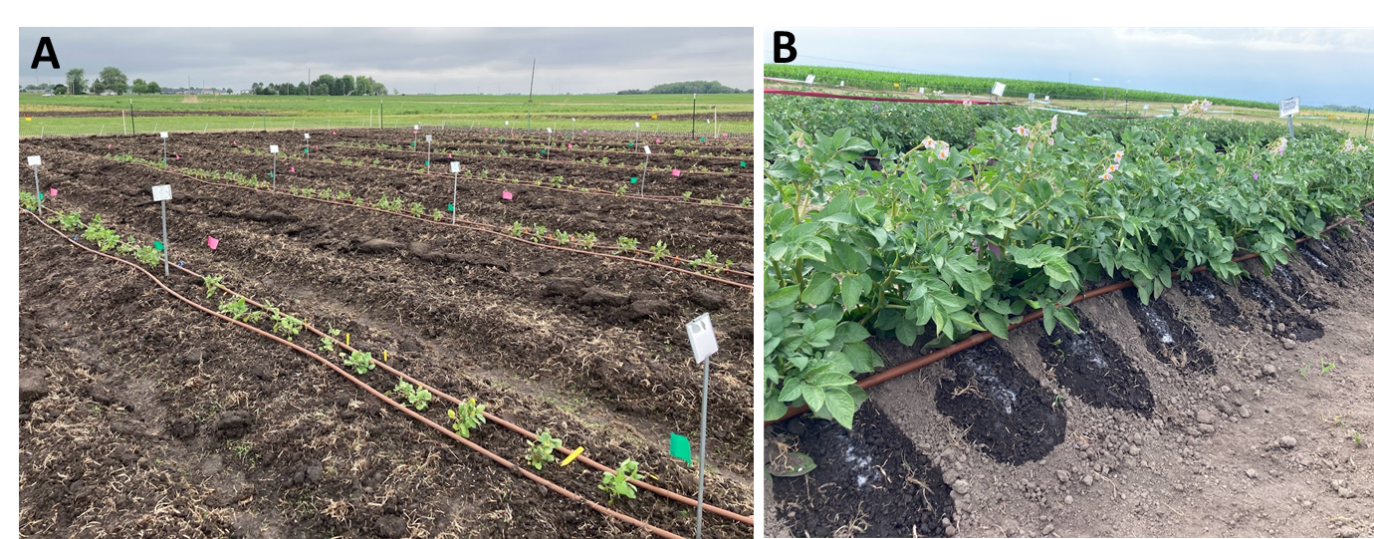
**

**Supplementary Figure 11. Field irrigation**

In all seasons, plots were watered as needed to prevent water limitation through drip irrigation piping on both sides of each hill in each plot. Tubing with holes was constructed on both sides of each hill and connected by a single point to a water pump dispensing equal water amounts to each plot when operating. The irrigation system is pictured at 5 DAT (A) and 55 DAT (B) in 2022.

**
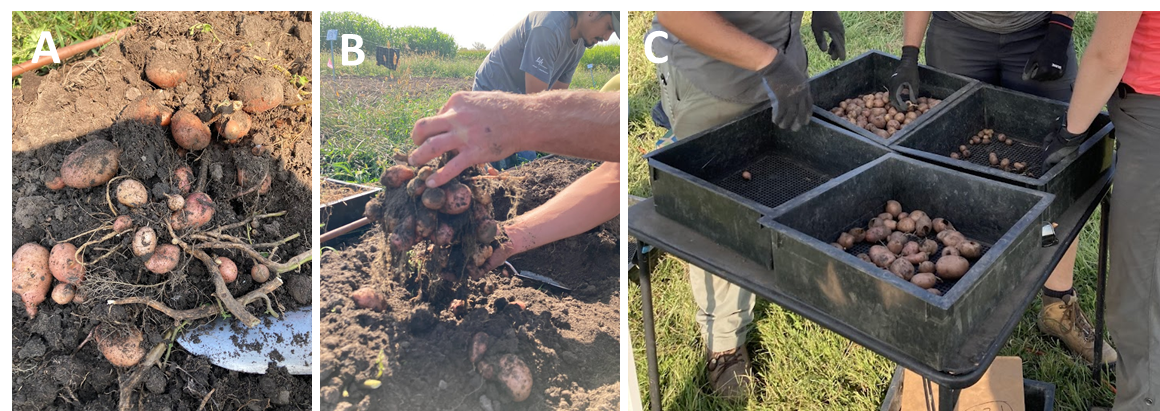
**

**Supplementary Figure 12. Tuber Harvest 2022**

All tuber harvests were conducted by hand in a single day. Above ground biomass was removed, tubers were dug up (A) and separated from roots (B). Tubers were cleaned, counted, weighed and sorted (C) to determine tuber counts and weights for tubers >2g (2019 and 2020) and >5g in 2022.

**Supplementary Table 3– Tuber nutritional contents 2020 and 2022
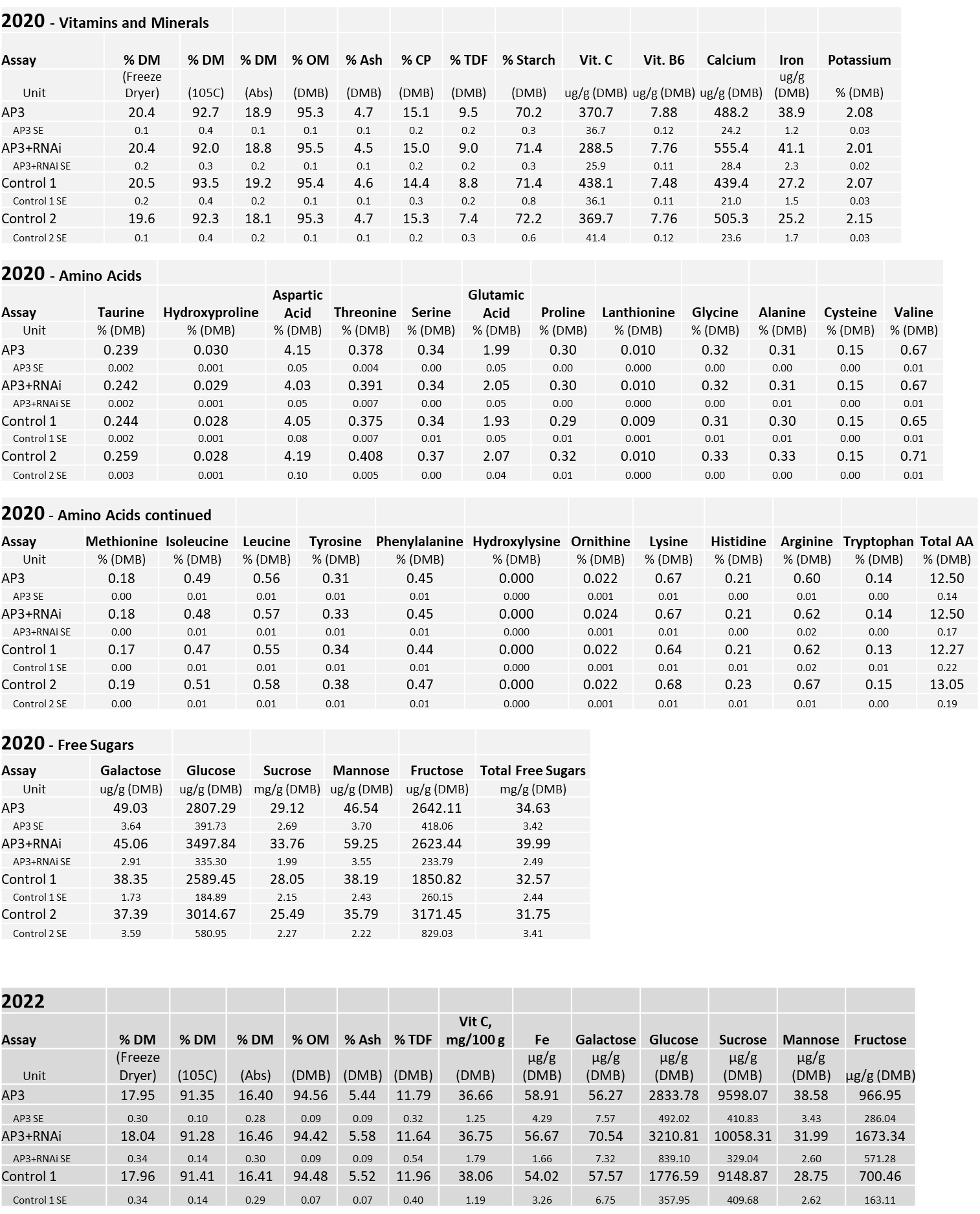
**

**Supplementary Table 4. Soil composition 2022**


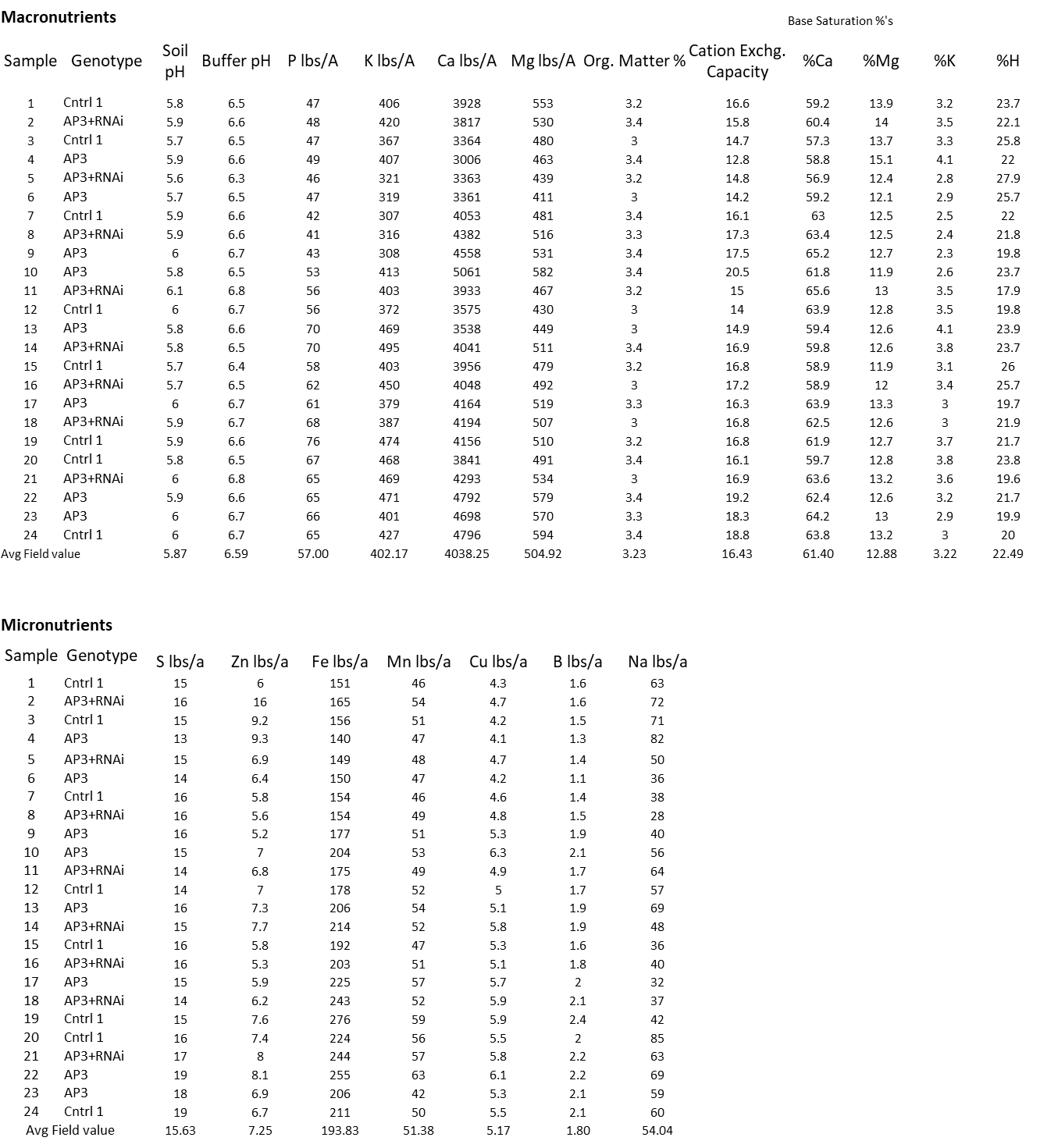


**Supplementary Table 5. Growth chamber settings for plants grown for metabolite analysis**

**Supplementary Table 6. RNA 2020 and 2022 P-values**

**Supplementary Table 7. Tuber mass 2020 and 2022 linear model ANOVA parameters**

**Supplementary Table 8. Diurnal assimilation and A’ statistics**

**Supplementary Table 9. V_cmax_, J_max_ and Γ statistics**

**18-Jul-22**
